# Cardiac *Bmi1*^+^ cells contribute to myocardial renewal in the murine adult heart

**DOI:** 10.1186/s13287-015-0196-9

**Published:** 2015-10-26

**Authors:** Iñigo Valiente-Alandi, Carmen Albo-Castellanos, Diego Herrero, Elvira Arza, Maria Garcia-Gomez, José C. Segovia, Mario Capecchi, Antonio Bernad

**Affiliations:** Cardiovascular Development and Repair Department, Spanish National Cardiovascular Research Center (CNIC), Madrid, Spain; The Heart Institute, Cincinnati Children’s Hospital Medical Center, Cincinnati, OH USA; Vivebiotech, San Sebastian, Spain; Immunology and Oncology Department, Spanish National Center for Biotechnology (CNB-CSIC), Madrid, Spain; Microscopy Unit, Spanish National Cardiovascular Research Center (CNIC), Madrid, Spain; Hematopoietic Innovative Therapies Division, Centro de Investigaciones Energéticas, Medioambientales y Tecnológicas (CIEMAT)- Centro de Investigaciones Biomédicas en Red de Enfermedades Raras (CIBERER), Madrid, Spain; Advanced Therapies Mixed Unit, Instituto de Investigación Sanitaria-Fundación Jiménez Díaz (IIS-FJD, UAM), Madrid, Spain; Howard Hughes Medical Institute University of Utah, Salt Lake City, UT USA

**Keywords:** Heart homeostasis, Stem cells, Bmi1, Cardiac progenitor cells

## Abstract

**Introduction:**

The mammalian adult heart maintains a continuous, low cardiomyocyte turnover rate throughout life. Although many cardiac stem cell populations have been studied, the natural source for homeostatic repair has not yet been defined. The Polycomb protein BMI1 is the most representative marker of mouse adult stem cell systems. We have evaluated the relevance and role of cardiac *Bmi1*^*+*^ cells in cardiac physiological homeostasis.

**Methods:**

*Bmi1*^CreER/+^;*Rosa26*^YFP/+^ (*Bmi1*-YFP) mice were used for lineage tracing strategy. After tamoxifen (TM) induction, yellow fluorescent protein (YFP) is expressed under the control of *Rosa26* regulatory sequences in *Bmi1*^*+*^ cells. These cells and their progeny were tracked by FACS, immunofluorescence and RT-qPCR techniques from 5 days to 1 year.

**Results:**

FACS analysis of non-cardiomyocyte compartment from TM-induced *Bmi1*-YFP mice showed a *Bmi1*^+^-expressing cardiac progenitor cell (*Bmi1*-CPC: B-CPC) population, SCA-1 antigen-positive (95.9 ± 0.4 %) that expresses some stemness-associated genes. B-CPC were also able to differentiate *in vitro* to the three main cardiac lineages. Pulse-chase analysis showed that B-CPC remained quite stable for extended periods (up to 1 year), which suggests that this *Bmi1*^+^ population contains cardiac progenitors with substantial self-maintenance potential. Specific immunostaining of *Bmi1*-YFP hearts serial sections 5 days post-TM induction indicated broad distribution of B-CPC, which were detected in variably sized clusters, although no YFP^+^ cardiomyocytes (CM) were detected at this time. Between 2 to 12 months after TM induction, YFP^+^ CM were clearly identified (3 ± 0.6 % to 6.7 ± 1.3 %) by immunohistochemistry of serial sections and by flow cytometry of total freshly isolated CM. B-CPC also contributed to endothelial and smooth muscle (SM) lineages *in vivo*.

**Conclusions:**

High *Bmi1* expression identifies a non-cardiomyocyte resident cardiac population (B-CPC) that contributes to the main lineages of the heart *in vitro* and *in vivo.*

**Electronic supplementary material:**

The online version of this article (doi:10.1186/s13287-015-0196-9) contains supplementary material, which is available to authorized users.

## Introduction

The adult mammalian heart was long considered a terminally differentiated organ with no capacity to replace aged or damaged cardiomyocytes (CM) [1]. This view was challenged by considerable evidence of low but intrinsic CM turnover in the adult mouse heart [2], although the contribution of adult CM turnover to heart homeostasis and the origin of the new cells remain unclear. There is compelling evidence that this low turnover rate throughout heart life mainly reflects the activity of a reservoir of cardiac stem cells (CSC) [3] that might reside in physiological niches [4, 5]. Dedifferentiation and division of pre-existing adult CM populations were recently proposed to contribute notably to heart turnover after myocardial infarction [6, 7].

Adult stem cells maintain and repair host tissues in adult organisms, and self-renewal, differentiation, and prevention of senescence of these cells are thus critical for tissue homeostasis. Adult resident cardiac stem/progenitors are defined primarily by the expression of cell surface markers such as c-KIT [8], SCA-1 [9, 10], ATP-binding cassette ABCG2 [11], PDGFRα [12] or combinations of these. The diversity of these findings has hindered a broad, unambiguous consensus for identification and molecular definition of endogenous CSC [3]. Several authors have emphasized the need for careful *in vivo* lineage tracing of CSC progeny to ascertain how this cell type contributes to CM replenishment during homeostasis or after myocardial injury [13–15]. Recent lineage tracing studies yielded interesting results. SCA-1 was reported to label a non-cardiomyocyte population in adult heart that clearly contributes to CM generation during homeostasis and normal aging (4.55 ± 0.87 %) [16]. In a *c-kit* lineage tracing study, c-KIT^+^ CSC appeared to make a small contribution to the generation of new CM (0.03 ± 0.008 %) in adult mouse heart [17]. Two additional lineage tracing studies, although not directly related to CSC regulation, should be mentioned. Malliaras *et al*. identified small non-myocyte cells termed cardioblasts (CdBs) that express sarcomeric α-actinin (38 %), α-MHC (39 %) and SCA-1 (55 %) but not c-KIT [18]. Activated CdBs apparently did not originate from hematogenous seeding, cardiomyocyte dedifferentiation, or mere expansion of a preformed progenitor pool; they, therefore, appear to arise by activation/differentiation of the endogenous CSC pool. Finally, a cardiac progenitor population defined as TIE-1^+^ CPC, a classical endothelial marker, is able to generate 70 % of the SCA-1^+^ intermediate perivascular progenitors that contribute (up to 3 %) to CM homeostatic turnover origin [19]. These findings reinforce the hypothesis that cardiac progenitor cells support the low CM turnover in the adult heart. Further genetic lineage tracing studies will help to elucidate the complex relationship between these partially different populations.

*Bmi1*, a member of the Polycomb repressive complex 1 (PRC1), is a transcription factor involved in many biological processes including embryonic development, organ formation, tumorigenesis, and stem cell stabilization and differentiation [20]. *Bmi1* has a crucial role during self-renewal and maintenance of hematopoietic, neural, intestinal, bronchioalveolar, pancreatic, prostate, lung and epithelial stem cells, as well as in the tongue and in rodent incisors [21–29]. There is little information on the role of *Bmi1* in the adult heart. Upregulation of *Bmi1* expression is cardioprotective against doxorubicin-induced damage [30]. A recent study demonstrated that, by controlling senescence, *Bmi1* expression in adult mouse CM is limiting dilated cardiomyopathy and heart failure [31]. Although a significant proportion of c-KIT^+^ human and porcine CSC expressed low BMI1 levels [32, 33], no specific study has addressed the functional relevance of this factor.

We hypothesized that adult cardiac progenitor cells may be characterized by high *Bmi1* expression, as in other adult stem cell compartments [21–29]. Using a validated lineage tracing strategy to track activity of the *Bmi1* locus, we show that the adult heart contains a resident non-cardiomyocyte population of *Bmi1*-expressing progenitor cells that constitute a fraction of the SCA-1^+^ population. *Bmi1*-CPC (B-CPC) show enriched expression of several multipotency and stemness markers, and their numbers increase throughout the lifetime of the mouse. B-CPC contribute significantly to the generation of *de novo* CM, endothelial and SM cells throughout life.

## Methods

### Transgenic mice and tamoxifen administration

*Bmi1*^CreER/+^;*Rosa26*^YFP/+^ (*Bmi1*-YFP) mice were generated by crossing the *Bmi1*^CreER/+^ strain with *Rosa26*^YFP/+^ reporter mice. Male and female *Bmi1*^CreER/+^;*Rosa26*^YFP/+^ double heterozygous mice received tamoxifen (TM; Sigma, Madrid, Spain) injections between postnatal days 30 (P30) and P60. TM was dissolved in corn oil (Sigma) to a final concentration of 20 mg/ml and mice received TM (i.p.) every 24 h on three consecutive days (9 mg per 40 g body weight). When indicated, *Bmi1*^CreER/+;^*Rosa26*^tomato/+^ (*Bmi1*-tomato), *Myh6*^MerCreMer/+^;*Rosa26*^YFP/+^ (*Myh6*-YFP) and *Bmi1*^CreER/+;^*Rosa26*^LacZ/+^ (*Bmi1*-LacZ) (Jackson Laboratory, Sacramento, California, United States) were used and TM-induced as above. All animal procedures conformed to EU Directive 86/609/EEC and Recommendation 2007/526/EC regarding the protection of animals used for experimental and other scientific purposes. The ethics committees of the Fundación Centro Nacional de Investigaciones Cardiovasculares (CNIC) and Centro Nacional de Biotecnología (CNB) approved animal studies.

### Immunodetection analysis

Heart immunohistochemistry was performed as previously described [7]. Specific yellow fluorescent protein (YFP) detection with anti-GFP antibody was confirmed by control immunofluorescence analysis of heart sections of TM-injected *Rosa26*^YFP/+^ mice and of non-induced *Bmi1*-YFP mice (*Bmi1*-YFP^NI^); no signal was observed for either (Fig. 2a). For immunodetection, sections were fixed in 2 % paraformaldehyde (PFA) and rinsed in PBS or PHEM buffer (25 mM Hepes, 10 mM EGTA, 60 mM PIPES, 2 mM MgCl_2_; all from Sigma). Slides were rinsed in blocking buffer (BB; 0.5 % porcine skin gelatin, 0.1 % bovine serum albumin; BSA; Sigma), incubated in 150 mM glycine (Merck, Madrid, Spain) (10 min, room temperature (RT)), followed by sodium borohydride (Sigma; 10 min) and finally in PBS with 0.1 % Triton X-100 (Sigma). Preparations were incubated with primary antibodies (see Additional file 1: Table S1) (1–3 h, RT), washed and incubated with the appropriate secondary antibody (1 h). Slides were incubated with Sytox Green and mounted in ProLong antifade reagent (both from Invitrogen, Madrid, Spain). Images were captured with a Leica SP5, Zeiss LSM 700 or LSM 780 coupled to a two-photon Spectra-Physics Mai Tai laser scanning confocal microscope and were assembled with ImageJ software (NIH). Processing, including assignment of pseudo-colors and changes in brightness, was applied uniformly to the entire image exclusively to equalize the appearance of multiple panels in a single figure. Immunocytochemistry was performed as above.

### LacZ staining

Adult cardiomyocytes were fixed in 0.25 % glutaraldehyde (Sigma; 5 min), washed with PBS twice (5 min), then incubated with wash buffer (0.1 M Na_2_HPO_4_:2H_2_O, 0.1 M NaH_2_PO_4_:H_2_0, 2 mM MgCl_2_, 0.11 % sodium deoxycholate, 0.2 % Igepal, 20 mM Tris–HCl pH 7.3) (3 min). Cells were incubated overnight with staining buffer (1 mg/ml X-Gal, 5 mM K_4_Fe(CN)_6_, 5 mM K_3_Fe(CN)_6_, followed by three washes with PBS (5 min).

### Cell isolation, culture and flow cytometry

Hearts were collected from *Bmi1*-YFP mice five days after TM induction, perfused with PBS to remove blood cells, and processed by enzymatic digestion using 0.1 % collagenase IV (Sigma) and 10 μg/ml DNAse (Roche, Madrid, Spain) (40 min, 37 °C). The resulting single cell suspension was passed through a 40 μm filter to remove debris. YFP^+^ cells were separated from total heart mass with a BD FacsAria II Special Order System cell sorter fitted with a 488 nm laser to excite YFP (collected in the 525/50 channel). To discriminate YFP^+^ from autoflorescent cells, a 488 nm laser was used to excite cells, followed by collection in the 585 channel (phycoerythrin). For flow cytometry analysis, cardiac cells from hearts of TM-induced *Bmi1*-YFP mice were incubated with the following primary and secondary antibodies as indicated: APC (allophycocyanin)-conjugated rat anti-c-KIT, APC-rat anti-SCA-1/Ly6a, biotin-rat anti-SCA-1/Ly6a, biotin-rat anti-CD45, biotin-rat anti-CD31 (all at 1:100; all from BD Pharmingen, Madrid, Spain), and streptavidin-Alexa Fluor 405 conjugate (1:500; Invitrogen). Labeled cells were examined with a BD Facs Canto II flow cytometer and data analyzed using Facs DIVA Software.

Purified YFP^+^ cells were cultured in Iscove’s modified Dulbecco’s medium (IMDM, Invitrogen) containing 10 % fetal bovine serum (ESCell FBS, Gibco, Madrid, Spain), 100 U/ml penicillin, 100 mg/ml streptomycin and 2 mM L-glutamine (all from Invitrogen), 10^3^ units ESGRO Supplement (Millipore, Madrid, Spain), 10 ng/ml EGF (epidermal growth factor; Sigma) and 20 ng/ml FGF (fibroblast growth factor; Peprotech, Rocky Hill, New Jersey, United States) (37 °C, 3 % O_2_, 5 % CO_2_). The SCA-1^+^ population was prepared by incubating the Lin^−^ primary cell suspension with rat anti-SCA-1/Ly6a biotin antibody (1:100: Abcam, Cambridge, United Kingdom), followed by isolation with Mouse Anti-Rat Kappa Microbeads (Miltenyi Biotec). In all cases, when preparing SCA-1^+^ cells, the CD45^+^ fraction was removed by indirect sorting using the MACS system and AUTOMACS technology (Miltenyi Biotec, Teterow, Germany). Cells were cultured in the same medium as YFP^+^ cells. Flow cytometry analysis of adult cardiomyocytes was performed in a BD LSR Fortessa TM using a neutral density filter 1.0. R1 mouse embryonic stem cells (ES), a gift from Dr. Miguel Torres (CNIC, Spain), were cultured on mitomycin C (Sigma)-inactivated murine embryonic fibroblasts as feeder cells in DMEM/Glutamax (Invitrogen) supplemented with 20 % ES-qualified FBS (Invitrogen), 10^3^ U/ml LIF (Millipore), 50 μM β-mercaptoethanol (Merck) and 1 % non-essential amino acids (Thermo, Madrid, Spain).

### Isolation and biochemical properties of adult mouse cardiomyocytes

Cardiomyocytes were isolated from hearts of TM-induced adult *Bmi1*-YFP mice. The heart was removed rapidly and retrograde-perfused under constant pressure (60 mmHg; 37 °C, 8 min) in Ca^2+^-free buffer containing 113 mM NaCl, 4.7 mM KCl, 1.2 mM MgSO_4_, 5.5 mM glucose, 0.6 mM KH_2_PO_4_, 0.6 mM Na_2_HPO_4_, 12 mM NaHCO_3_, 10 mM KHCO_3_, 10 mM Hepes, 10 mM 2,3-butanedione monoxime, and 30 mM taurine. Digestion was initiated by adding a mixture of recombinant enzymes (0.2 mg/ml Liberase Blendzyme (Roche), 0.14 mg/ml trypsin (Invitrogen), and 12.5 μM CaCl_2_ to the perfusion solution). When the heart became swollen (10 min), it was removed and gently teased into small pieces with fine forceps in the same enzyme solution. Heart tissue was further dissociated mechanically using 2, 1.5, and 1 mm-diameter pipettes, until all large heart tissue pieces were dispersed. The digestion buffer was neutralized with stopping buffer containing 10 % FBS and 12.5 μM CaCl_2_. Cardiomyocytes were pelleted by gravity (20 min), the supernatant aspirated and cells resuspended in the perfusion solution containing 5 % FBS and 12.5 μM CaCl_2_. The calcium concentration was increased by gradually adding CaCl_2_ from 62 μM to 1 mM final concentration. Cardiomyocytes were plated in culture dishes precoated with 0.5 mg/ml mouse laminin (BD Biosciences) in PBS (1–2 h, RT). Plating medium was Medium 199 Hank’s (Invitrogen), 0.25 % BSA (Sigma), 22 mM NaHCO_3_, 0.05 % FBS (Sigma), 0.001 % ITS (insulin-transferrin-selenium (Gibco), 10 mM 2,3-butanedione monoxime and 25 μM blebbistatin. After 2 h, cardiomyocytes were fixed with 2 % PFA or were used for the *in vitro* calcium transient studies. To detect YFP^+^ cardiomyocytes (YFP^+^ CM), we used a confocal microscope LSM 780 upright scanning system (Zeiss) equipped with a W 20X Plan-APOCHROMAT dipping objective (numerical aperture (NA) = 1.0). YFP^+^ CM were detected using the 514 nm laser to excite YFP (acquired in the 535 channel). A transmitted light detector (T-PMT) was used to screen cardiac cell morphology. We captured and stored YFP cardiomyocyte images based on cell coordinates before Fluo-4 labeling.

For Fluo-4 AM labeling, we prepared a stock solution of 1 mM Fluo-4 AM (Invitrogen) in DMSO with an equal volume of 20 % Pluronic F-127 DMSO (1:1 ratio); the working concentration was 1 μM. Fluo-4 AM was added to DMEM supplemented with 100 U/ml penicillin, 100 mg/ml streptomycin and 2 mM L-glutamine; cells were incubated in the dark (20–30 min). We washed the cells and added fresh DMEM without phenol red (Sigma) and images were acquired by confocal microscopy as for Ca^2+^ fluorescence. Fluo-4 was excited with the 488 nm line of an argon laser and 505 nm signal emissions were collected. Images were captured in a time series (xyt, pixel dwell 1.58 μs) and 2D images (512 × 512 lines) were obtained and stored for offline analysis.

### Primary culture of neonatal rat cardiomyocytes

Hearts from one-day-old Wistar rats were minced to 1 mm^2^ and digested with 0.05 % trypsin (Invitrogen) in Hank’s balanced salt solution (Sigma)(37 °C, 40 min). The fragments were digested with 0.1 % collagenase (class II, Worthington Biochemical, Lakewood, New Jersey, United States). Single-cell suspensions were prepared by mechanical pipetting. Cells were passed through a 40 μm filter and preplated in DMEM (Invitrogen) supplemented with 10 % FBS, 100 U/ml penicillin, 100 mg/ml streptomycin and 2 mM L-glutamine (2 h, 37 °C). Newborn rat cardiomyocytes (NBRC) were collected and seeded on coverslips precoated with gelatin (Invitrogen) and fibronectin (BD Biosciences) in a final medium containing DMEM, M-199 (Gibco) at 4:1, 10 % horse serum (Sigma), 5 % FBS, 100 U/ml penicillin, 100 mg/ml streptomycin, 2 mM L-glutamine and 1 μg/ml cytosine α-D-arabinofuranoside (Sigma). Neonatal mouse cardiomyocytes were isolated as indicated for adult mice, using M-199 medium supplemented with 0.05 % FBS, 0.001 % ITS and 25 μM blebbistatin.

### Explant cultures

Explants were prepared from hearts of eight-week-old TM-induced *Bmi1*-YFP mice, as described [34], and cultured in IMDM containing 20 % embryonic stem cell-screened FBS (Hyclone, GE Healthcare Life Sciences, Madrid, Spain), 100 U/ml penicillin, 100 mg/ml streptomycin and 2 mM L-glutamine (37 °C, 5 % CO_2_).

### B-CPC differentiation potential

To evaluate spontaneous endothelial/smooth muscle differentiation potential of *Bmi1*-CPC, cells were obtained from eight-week-old TM-induced *Bmi1*-YFP mouse hearts and seeded on gelatin-coated plates as above. After seven to ten days, plates were fixed with 2 % PFA in PHEM buffer (15 min, RT) and processed for immunocytochemistry. For cardiomyocyte differentiation, B-CPC cardiospheres were plated on a monolayer of NBRC or adult transgenic mouse GFP^+^ CM derived from the *beta-actin* GFP mouse strain (Jackson Laboratory). For B-CPC/adult mouse CM co-culture, we used M-199 medium, 0.05 % FBS, 0.001 % ITS and 25 μM blebbistatin. After four to five days co-culture, cells were fixed with 2 % PFA in PHEM buffer (15 min, RT) and processed for immunocytochemistry.

### Bone marrow transplant

To generate bone marrow (BM) chimeras, eight to ten week-old C57BL/6 *Bmi1*-YFP mice were TM-induced, lethally irradiated (one dose each of 4.75 and 4.5 Gy, separated by 24 h) and then transplanted (i.v.) with 10^7^ whole BM cells isolated age-matched C57BL/6 *Act*-RFP mice [35]. At two months post-TM induction (54 days after BM transplant), after confirmation of full chimerism, transplanted *Bmi1*-YFP mouse hearts were digested and analyzed as above.

### RT-qPCR and genomic PCR analysis

RNA was extracted from hearts of eight-week-old TM-induced *Bmi1*-YFP mice, or from the indicated subpopulations (*Bmi1*-CPC and SCA-1 CPC) purified using the sorting strategy described above, with a Cells-to-CT kit (Ambion, Thermo, Madrid, Spain). RNA from ES cells was prepared as previously described [36]. Complementary DNA was obtained by reverse transcription with the High Capacity cDNA Reverse Transcription Kit (Applied Biosystems, Madrid, Spain). cDNAs were analyzed by real time PCR using the Power SYBR Green PCR Master Mix (Applied Biosystems). Amplification, detection and data analysis were carried out with an ABI PRISM 7900HT Sequence Detection System. The crossing threshold values for individual mRNAs were normalized to *GusB* expression for mRNA. Changes in mRNA expression were denoted as the *x-*fold change relative to the control. (See Additional file 2: Table S2 for primers used).

We used genomic PCR to detect recombined and *Rosa26-*YFP alleles, with primers 5′-AAAGTCGCTCTGAGTTGTTAT, 5′-AAGACCGCGAAGAGTTTGTC and 5′-AGCTC CTCGCCCTTGCTCACCATG [17]. PCR conditions were 96 °C for 2 min to separate strands, followed by 34 amplification cycles (96 °C for 30 s, 56 °C for 30 s, 72 °C for 30 s) and a 5 min elongation step at 72 °C. The specific PCR product (320 bp) derived from the floxed allele is detected in all transgenic *Bmi1*-YFP and *Myh6* -YFP mice, but the diagnostic fragment (550 bp) associated with the floxed-out allele is only detectable in the *Myh6* -YFP CM-enriched fraction post-TM induction [37].

### Statistical analysis

Statistical analysis was performed with Prism 5.0 (GraphPad Software). Significance between groups was evaluated in all experiments as detailed in the figures. A value of P < 0.05 was considered significant. All replicates considered are biological replicates.

## Results and Discussion

### Murine SCA-1^+^ CPC population expresses *Bmi1* at similar levels to embryonic stem cells

We hypothesized that adult cardiac progenitors would show high *Bmi1* expression, as it has been previously defined in other adult stem cell systems [21–29, 38]. The cardiac SCA-1^+^ population (SCA-1^+^ CPC) contains cardiac progenitor cells [10]. We initially compared expression of *Bmi1* and other stemness-related genes in SCA-1^+^ CPC and embryonic stem cells (ES), and found low but comparable *Bmi1* levels in both populations (see Additional file 3: Figure S1A). *Bmi1* expression in these cells was also very sensitive to oxygen culture conditions and passage number (see Additional file 3: Figure S1B). These preliminary studies confirmed *Bmi1* expression in SCA-1^+^ CPC at levels comparable to ES, and established culture conditions appropriate for maintenance of *Bmi1* expression. We therefore studied whether *Bmi1* levels corresponded to the entire SCA-1^+^ CPC population or whether defined subpopulations were associated with its expression.

### High *Bmi1* expression defines a self-maintained population in the adult mouse heart

**Fig. 1:**
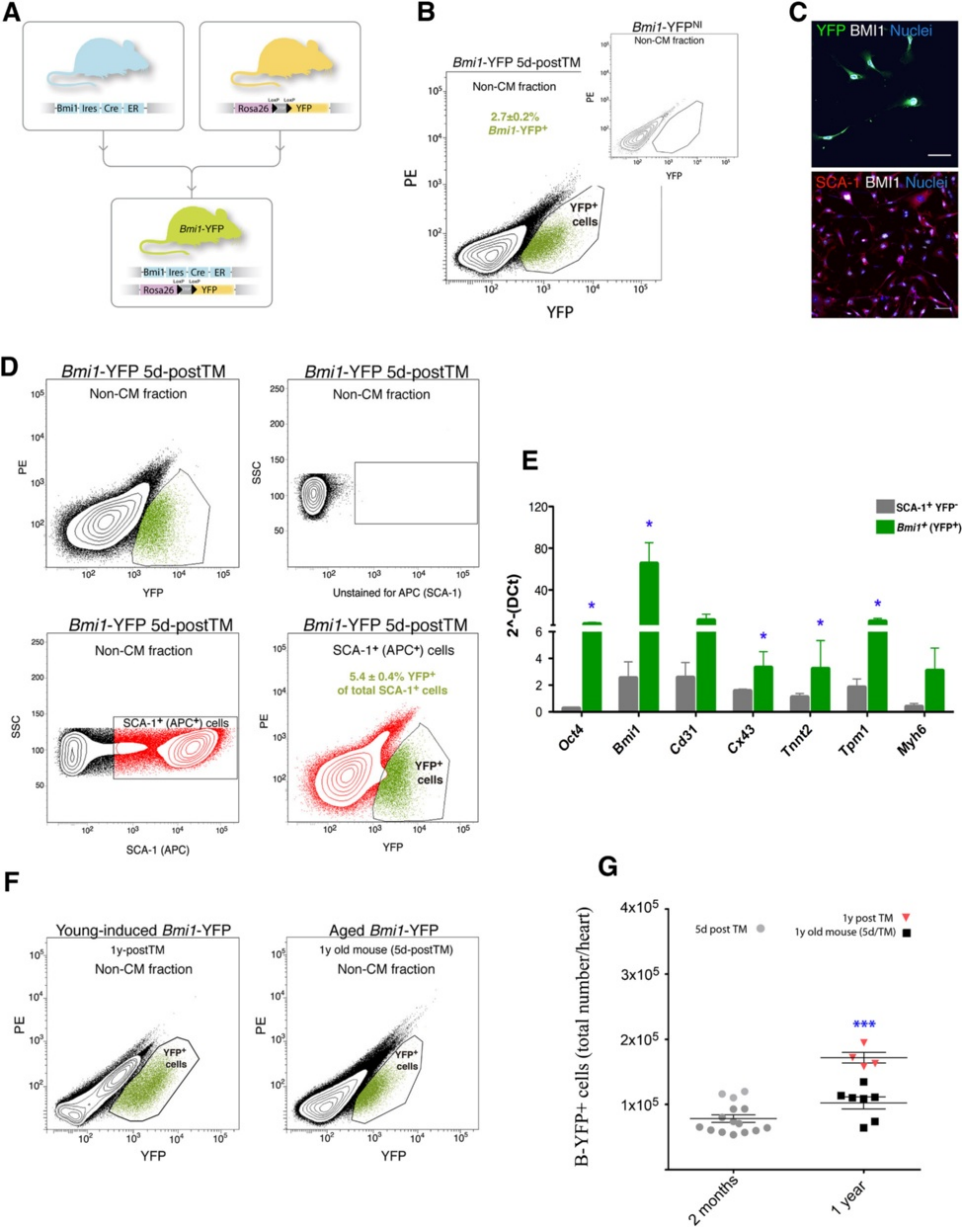
Characterization and evolution of B-CPC throughout *Bmi1*-YFP mouse lifespan. **a** Generation of *Bmi1* ^CreER/+^;*Rosa26* ^YFP/+^ (*Bmi1*-YFP) mice. **b** Detection of the YFP^+^ fraction (2.7 ± 0.2%, *n* = 21) of freshly isolated mononuclear non-cardiomyocyte heart cells from *Bmi1*-YFP mice, analyzed five days post TM induction (5d-postTM); *inset*, *Bmi1*-YFP^NI^ (non-induced) and TM-induced *Rosa26* ^YFP/+^ negative controls. Data shown as mean ± SEM. PE, phycoerythrin. **c** Immunofluorescence analysis of BMI1 and SCA-1 in freshly isolated YFP^+^ cells. Bars, 50 μm. **d** The B-CPC population is a subset of the SCA-1^+^ population (5.4 ± 0.4 %, *n* = 18). The plots show from (*left to right* and top *up to bottom*) the YFP^+^ fraction of *Bmi1*-YFP hearts 5d-postTM, the negative control from SCA-1 staining in the non-CM fraction, staining for SCA-1, and the fraction of the YFP^+^ SCA-1^+^ population. Data represented as mean ± SEM. PE, phycoerythrin, SSC, side scatter. **e** RT-qPCR of freshly sorted *Bmi1* ^+^ (YFP^+^) and SCA-1^+^ YFP^−^ cells (*n* = three replicates; two to three mice per replicate). Data shown as mean ± SD. P values were calculated by paired Student’s t-test. * P < 0.05, ** P < 0.01. **f** Analysis of the YFP^+^ compartment in *Bmi1*-YFP mice at one year post-TM induction (1y-postTM) (*left*) (5.8 ± 0.74 %) and analysis of B-CPC (YFP^+^) in one-year-old mice 5d-postTM (*right*) (2.2 ± 0.22 %). PE, phycoerythrin, SSC, side scatter. **g** YFP^+^ cell number at 5d-postTM in young mice (two-month-old; *n* = 15), in mice 1y-postTM induction at six to eight weeks of age (*n* = 4), and in one-year-old mice at 5d-postTM (*n* = 7). Data shown as mean ± SEM. P values were calculated by unpaired Student’s t-test with Welch’s correction, compared to 5d-postTM young mice. * P < 0.05, *** P < 0.0001

To evaluate the role of *Bmi1* in the biology of cardiac progenitor cells *in vivo*, we conducted a lineage tracing analysis in mice expressing TM-inducible Cre driven by the *Bmi1* locus [23, 24]. Cre-mediated recombination in *Bmi1*^CreER/+^;*Rosa26*^YFP/+^ (six to eight week-old) double heterozygous mice (*Bmi1*-YFP mice; Fig. 1a) was induced by TM administration. After enzyme digestion of the heart, non-myocyte cell compartment was separated and analyzed by FACS (see Methods). Flow cytometry of non-cardiomyocyte cells at five days post-TM induction (5d-postTM) identified a YFP^+^ population (*Bmi1*-expressing cells) (2.7 ± 0.2 %; 7.9 x 10^4^ ± 5.9 x 10^3^ YFP^+^ cells/heart; Fig. 1b) that was not detected in age-matched non-induced controls (*Bmi1*-YFP^NI^) or induced *Rosa26*^YFP/+^ mice (Fig. 1b, inset). Immunofluorescence analysis of freshly sorted YFP^+^ cells confirmed co-expression with BMI1 and SCA-1 (Fig. 1c).

We used flow cytometry to characterize non-CM YFP^+^ cells derived from *Bmi1*-YFP hearts at 5d-postTM (see Additional file 4: Figure S2A); most were SCA-1^+^ (95.9 ± 0.4 %, *n* = 16), although they made up only 5.4 ± 0.4 % of the total SCA-1^+^ population (Fig. 1d). These non-CM YFP^+^ cells were mainly CD45^−^ (<1 %) and c-KIT^−^ (<0.5 %) but CD31^+^ (94 %) (see Additional file 4: Figure S2A). We used RT-qPCR to compare the gene expression profile of the sorted YFP^+^ population with that of the closely related SCA-1^+^ YFP^−^ cardiac population (S-YFP^−^). The YFP^+^ population showed higher expression of stemness-associated genes and transcripts related to muscle contractility (Fig. 1e). These results suggest that *Bmi1*^+^ cells are probably a heterogeneous population, we cannot currently distinguish whether this heterogeneity is due to non-progenitor cells or to different progenitor populations.

Analysis of the YFP^+^ compartment (the progeny of *Bmi1*^*+*^ cells) at one-year post-TM induction showed that this population increased during this period (2 x 10^4^ ± 4 x 10^3^ cells/heart; Fig. 1f, g), with an expression profile similar to that of 5d-postTM cells (see Additional file 4: Figure S2B). Analysis after conventional TM induction (5d-postTM) of aged (one-year-old) mice showed the presence of the YFP^+^ population (Fig. 1f, g).

These results suggest that the *Bmi1*^+^ non-myocyte population (B-CPC) is maintained throughout the mouse lifespan and is apparently not in equilibrium with more primitive precursors that would dilute initial labeling.

### B-CPC spatial distribution and differentiation potential

**Fig. 2:**
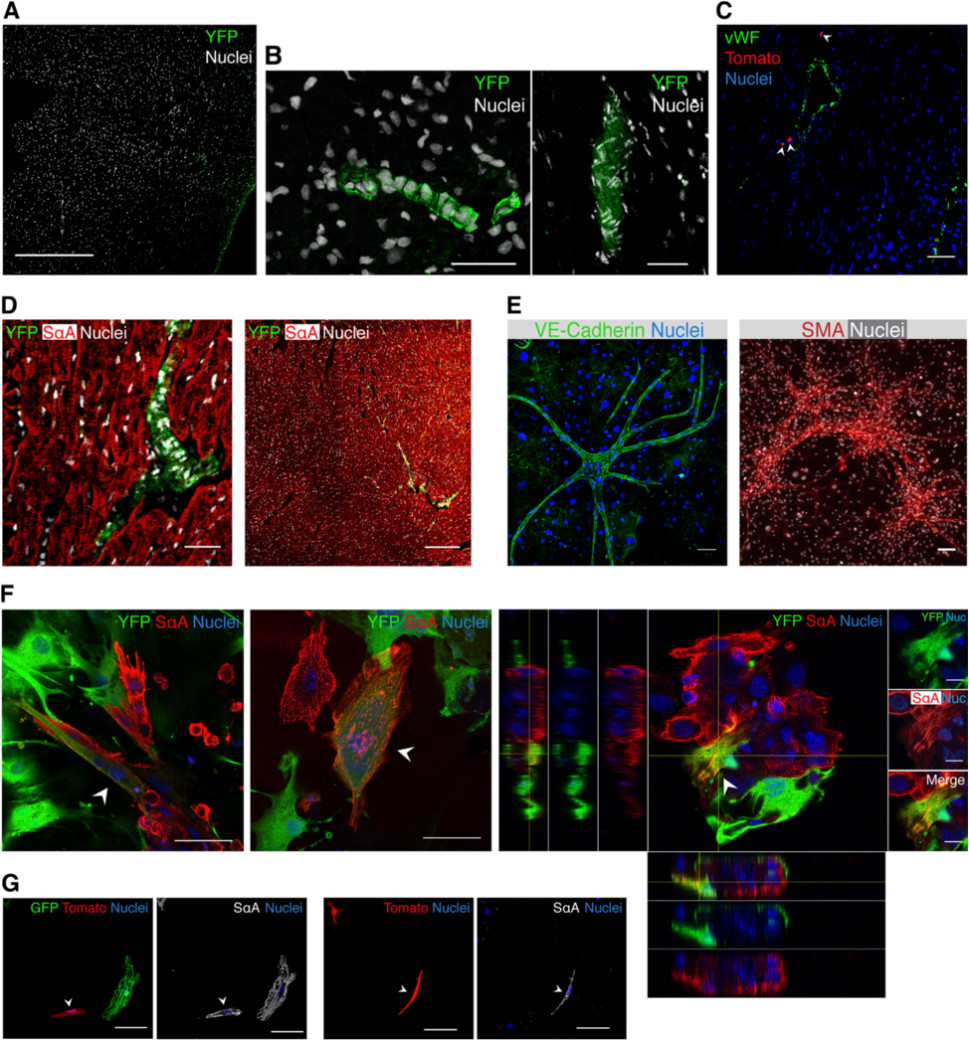
B-CPC tissue distribution and *in vitro* differentiation capacity. **a** Specific GFP immunohistochemistry of negative control heart section; *Bmi1*-YFP^NI^ or TM-induced *Rosa26* ^YFP/+^ mice showed no specific staining. Bar, 200 μm. **b** YFP labels highly packed niche-like structures (NLS) in the myocardium wall of *Bmi1*-YFP heart 5d-postTM induction. Bar, 20 μm. **c**, **d** These niche-like structures are widely distributed throughout the organ and show preferential perivascular (*arrowheads*) (**c**) and intersarcomeric (**d**) localization. Bar in (**c**), 50 μm. Bars in (**d**), 100 μm (*left*), 200 μm (*right*). **e** Representative images showing *in vitro* vascular differentiation of sorted YFP^+^ cultures, which contain cells positive for VE-cadherin (*left*) and SMA (smooth muscle actin) (*right*); DAPI, blue. Bars, 200 μm. **f** YFP^+^ cells co-cultured *in vitro* for four to five days with neonatal rat CM differentiate to the cardiomyocyte lineage, and co-localize with sarcomeric α-actinin (SαA); the orthogonal projection is shown (*right composite panel*). *Arrowheads* show the differentiated YFP^+^ cells. Bars, 50 μm. **g** *Bmi1*-tomato^+^ cells co-cultured *in vitro* with adult GFP-CM from β*-actin* GFP mice begin to express SαA (white). Images (*left*) show a tomato^+^ cell (*arrowhead*) expressing SαA next to a GFP^+^ CM. Images (*right*) show two tomato^+^ cells (no GFP^+^ CM on the picture), one of which expresses SαA (*arrowhead*). Bars, 20 μm. *B-CPC Bmi1*-expressing cardiac progenitor cells, *YFP* yellow fluorescent protein, *TM* tamoxifen, *VE* vascular endothelial, *CM* cardiomyocytes, *SαA* sarcomeric α-actinin

To locate *Bmi1*^+^ cells in mouse heart, we used mainly a *Bmi1*-YFP transgenic model and complemented the study with *Bmi1*-tomato mice for additional information. GFP immunostaining from 5d-postTM *Bmi1*-YFP hearts showed broad YFP distribution not observed in negative controls (Fig. 2a). B-CPC cells were located in variable-sized clusters of compact cells with cramped nuclei (Fig. 2b) scattered throughout heart sections, with preferential perivascular (Fig. 2c) and inter-sarcomeric localization (Fig. 2d). We found broad distribution of B-CPC cells both in atria and ventricles (see Additional file 5: Figure S3). GFP immunostaining of cardiac explants [39] from 5d-postTM *Bmi1*-YFP mice showed that a large proportion of the bright, rounded cells that migrated on the fibroblast-like layer were YFP^+^. In orthogonal projections, the rounded BMI1^+^ cells selectively occupied the upper layer (see Additional file 6: Figure S4A). Like their freshly sorted counterparts 5d-postTM, explanted YFP^+^ cells expressed BMI1 (see Additional file 6: Figure S4B). This result suggests that *Bmi1*^+^ cells are related to previously reported populations now being evaluated in clinical trials [40, 41].

Cardiac progenitor cells are able to differentiate to the main heart lineages. Although several methods have been tested for *in vitro* differentiation, results for terminal differentiation with sarcomeric structure and beating cardiomyocytes are poor [17, 42, 43]. We examined the differentiation potential of B-CPC *in vitro*, based on our previous work with SCA-1^+^ CPC [44]. Only a small percentage of sorted YFP^+^ B-CPC developed sporadic microvascular networks composed of VE (vascular endothelial)-cadherin^+^ and SMA^+^ (smooth muscle actin) cells (Fig. 2e), indicating that although B-CPC initially express CD31, they are not committed to the vascular lineage. It has been reported that hematopoietic stem cells, in different stages of development, or cardiac progenitor cells are characterized by the expression of endothelial markers, such as CD31 and Tie2 [19, 40, 45]. Moreover, CD34 expression has been found in various stem cell systems [46]. These findings indicate that these proteins have various cellular functions and are not restricted to mature endothelium [47].

Co-culture of neonatal rat CM with B-CPC (*Bmi1*-YFP mice) promoted their cardiac differentiation, with co-expression of sarcomeric α-actinin (SαA) and YFP (Fig. 2f). Although the frequency of mononucleated YFP^+^ cells that co-stained for SαA was low, orthogonal projection of these cells confirmed co-expression (Fig. 2f, right). Results were similar when we co-cultured B-CPC (*Bmi1*-tomato mice) with adult mouse GFP^+^ CM (*β-actin* GFP mice) to discard fusion events. Although we did not detect marked mature differentiation, some tomato^+^ cells initiated a SαA expression and CM transition program without fusion, detectable by double GFP^+^tomato^+^ staining (Fig. 2g). These data suggest that the B-CPC population contains progenitors able to contribute all three main heart lineages.

### B-CPC contribute to adult cardiomyocyte turnover

**Fig. 3:**
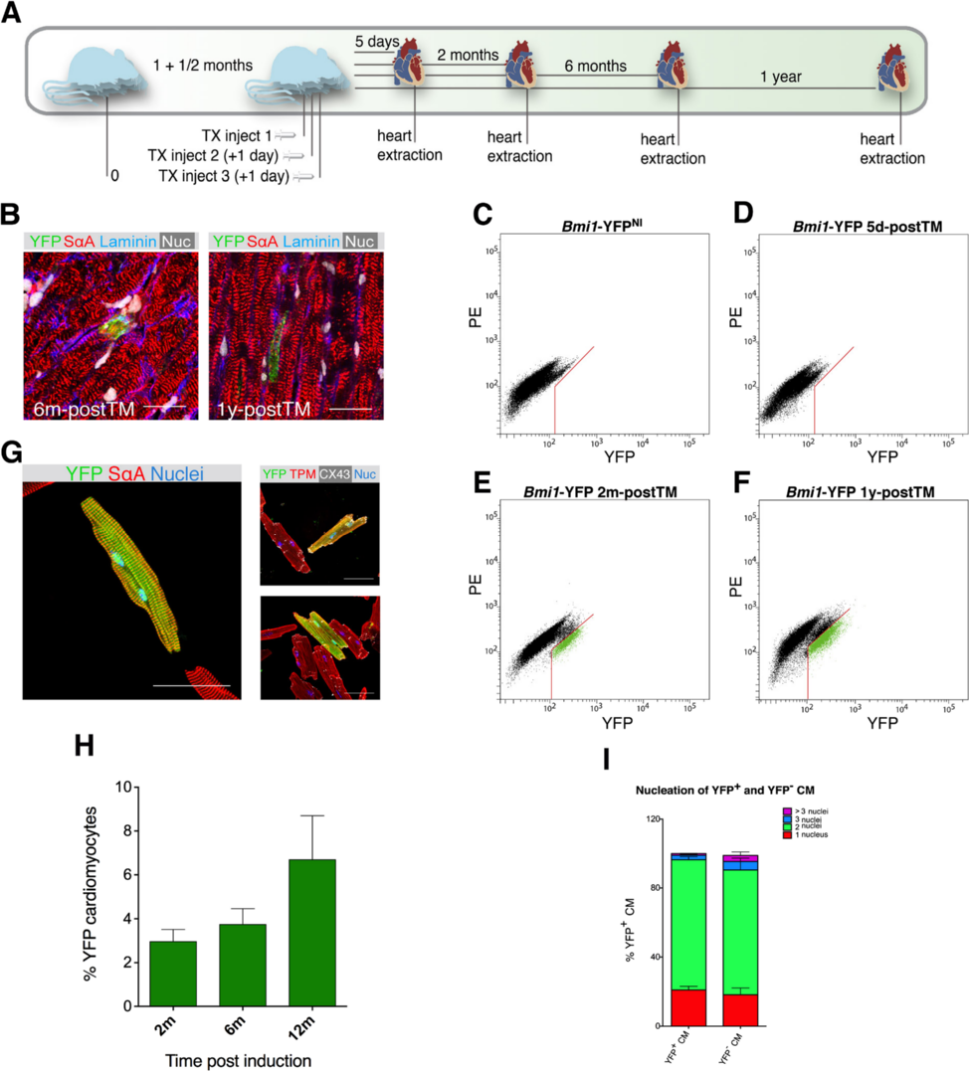
*In vivo* contribution of B-CPC to homeostasis in the adult mammalian heart. **a** Timeline for B-CPC and progeny analysis. **b** Immunohistochemistry of mature YFP^+^ SαA-positive CM surrounded by laminin, at 6 (*n* = 2) and 12 months (*n* = 2) post-TM induction, were scattered throughout the heart. Bars, 20 μm (left), 50 μm (right). **c**-**f** Flow cytometry of total adult CM from (**c**) *Bmi1*-YFP^NI^ (*n* = 3), (**d**) *Bmi1*-YFP 5d-postTM (*n* = 8), (**e**) *Bmi1*-YFP at two months (*n* = 5) and (**f**) *Bmi1*-YFP mice one year post-TM induction (n = 6). **g** Expression of contractility proteins in YFP^+^ and YFP^−^ CM; SαA, tropomyosin (TPM) and connexin 43 (CX43). Bars, 50 μm. **h** Number of YFP^+^ CM, measured by flow cytometry and immunocytochemistry, increases from 2 to 12 months post-TM induction, 3 ± 0.6 % at 2 months (2m; *n* = 7), 3.8 ± 0.7 % at 6 months (6m; *n* = 3) and 6.7 ± 2 % at 12 months (12m; *n* = 9). Data shown as mean ± SEM. P value was calculated by unpaired Student’s t-test with Welch’s correction. * P < 0.05, ** P < 0.01. **i** Analysis of ploidy in freshly plated adult CM. To count, plated CM were immunostained with YFP and SαA antibodies and scored for DAPI-stained nuclei. A mean of 1,000 CM were scored for each mouse (*n* = 6). Data shown as mean ± SEM. *B-CPC Bmi1*-expressing cardiac progenitor cells, *YFP* yellow fluorescent protein, *SαA* sarcomeric α-ctinin, *CM* cardiomyocytes, *TM* tamoxifen

To assess the role of B-CPC in homeostatic conditions, we traced YFP^+^ cells in the adult heart throughout the TM-induced *Bmi1*-YFP mouse lifetime (Fig. 3a). Immunohistochemical analysis of *Bmi1*-YFP heart sections from 2 to 12 months post-TM administration showed YFP^+^ CM, which co-localized with SαA staining (Fig. 3b). To confirm these results and further evaluate the appearance of YFP^+^ CM, we isolated and plated the enriched CM fraction for dual analysis by flow cytometry and immunostaining.

**Fig. 4:**
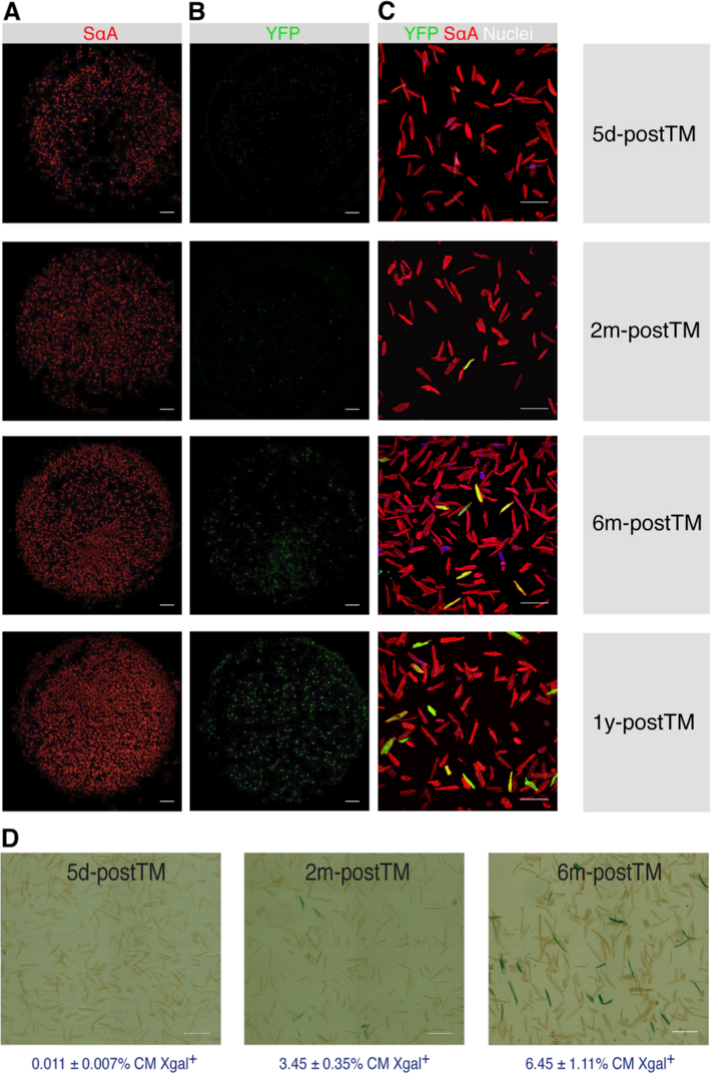
Contribution of YFP^+^ cardiomyocytes to heart homeostasis. **a**-**c** Immunofluorescence of freshly-isolated CM from *Bmi1*-YFP mice TM-induced at six to eight weeks of age, throughout mouse lifetime at days (5d), 2 months (2m), 6 months (6m) and 12 months (12m) post-TM induction. SαA in red (**a**), anti-YFP staining in green (**b**) and CM at higher magnification (**c**); DAPI, blue. Bars in (**a**), (**b**), 1,000 μm; (c), 200 μm. **d** LacZ^+^ CM in adult *Bmi1*-LacZ mice 5 days (*n* = 4), 2m (*n* = 2) and 6m (*n* = 2) post-TM induction. Bars, 200 μm. Data shown as mean ± SEM. *YFP* yellow fluorescent protein, *CM* cardiomyocytes, *TM* tamoxifen, *SαA* sarcomeric α-actinin

No YFP expression was detected in the CM-enriched population shortly after induction (5d-postTM), either by cytometry (<1 YFP^+^ CM in 1.35 x 10^5^ total cardiomyocytes or < 0.0022 ± 0.0016 %) (Fig. 3d) or in plated preparations (Fig. 4). To confirm the specific *Bmi1* expression in the YFP^+^ sorted fraction 5d-postTM and not in the CM compartment, we performed RT-qPCR analysis, which showed preferential *Bmi1* expression mainly in the YFP^+^ non-CM fraction (>15-fold; see Additional file 7: Figure S5A, top). No BMI1 protein expression was detected by western blot in the sorted YFP^−^ non-CM compartment or in the enriched CM fraction (see Additional file 7: Figure S5A, bottom). To further confirm the lack of *Rosa26* locus recombination in CM after TM induction, we isolated the CM-enriched fraction from *Bmi1*-YFP mice 5d-postTM, and analyzed the structure of the transgenic *Rosa26* locus by PCR. There was no detectable recombination in the CM-enriched fraction from *Bmi1*-YFP mice compared with positive and negative controls (see Additional file 7: Figure S5B).

In addition, we analyzed Cre expression by immunocytochemistry in plated CM; no Cre expression was detected in the enriched CM fraction of *Bmi1*-YFP mice compared to the CM fractions of *Myh6*-YFP or WT mice (see Additional file 7: Figure S5C). This result strongly suggests that the cardiac *Bmi1*-expressing fraction does not include a minor subpopulation of adult CM with specific dedifferentiation/proliferation potential [6]. Given the wide variety of *Bmi1* functions, we cannot currently rule out *Bmi1* expression in intermediate progenitors or trace levels in differentiated cells. The *Bmi1*CreERT construct is an inducible transgenic model in which a specific Cre expression threshold is necessary to trigger *Rosa26* locus recombination [48]*.* Although we cannot completely exclude basal *Bmi1* expression in CM, we confirmed that the lineage-tracing results were not due to ectopic Cre expression by CM.

**Fig. 5:**
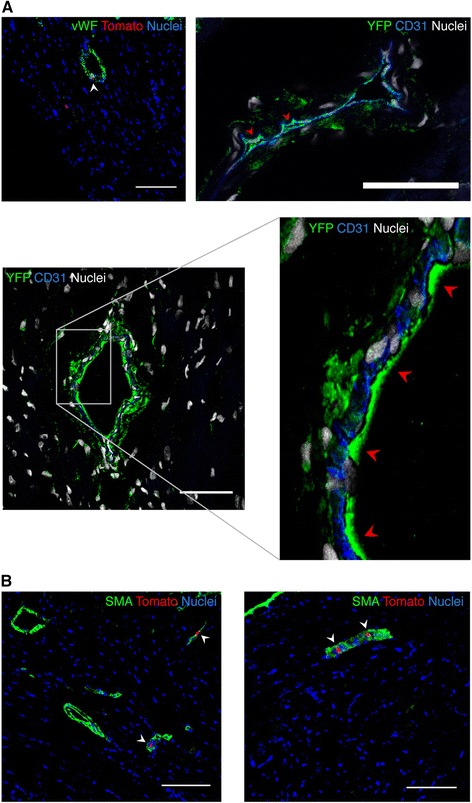
Differentiation to smooth muscle and endothelial lineages. **a** Confocal microscopy of differentiated *Bmi1*-tomato^+^-derived cells expressing von Willebrand factor (vWF, *top left*, *white arrowhead*) or differentiated *Bmi1*-YFP^+^-derived cells expressing CD31 (*top right; bottom, red arrowheads*) lining the vessel lumen; tomato^+^/YFP^+^ cells at the luminal surface of vessels during endothelial differentiation co-localize with vWF or CD31 (*arrowheads*). Bars, 100 μm (*top left*), 20 μm (*top right*), 50 μm (*bottom*). **b** Vascular differentiation of *Bmi1*-expressing cells to the smooth muscle lineage; *arrowheads* highlight tomato co-localization with SMA. Bars, 100 μm. *YFP* yellow fluorescent protein, *SMA* smooth muscle actin

Flow cytometry analysis of CM from *Bmi1*-YFP hearts at 2 to 12 months showed the presence of YFP^+^ CM (Fig. 3e, f), as confirmed by immunocytochemistry of plated CM (Fig. 4a-c). YFP^+^ and YFP^−^ CM showed no relevant differences in expression of the contractility proteins tropomyosin and SαA or of CX43 (Fig. 3g), or in contractility or transient Ca^2+^ efflux (see Additional file 8: Figure S6). The proportion of YFP^+^ CM increased over the analysis period (2–12 months) from 3 ± 0.6 % to 6.5 ± 1.3 % (Fig. 3h), with the same nucleation pattern as the YFP^−^ CM (Fig. 3i), which are mainly binucleated (80 %). We also observed clear YFP staining within vessels that co-localized with von Willebrand factor (vWF) or CD31 in cells lining the vessel lumen (Fig. 5a) as well as with SMA (Fig. 5b), which suggest a contribution to endothelial and smooth muscle lineages.

To validate these results independently, we examined the *Rosa26*LacZ reporter mice. Xgal staining of freshly isolated CM from adult hearts confirmed lack of leakage in non-induced *Bmi1*-LacZ and a very low level (<0.011 ± 0.007 %) in 5d-postTM induced *Bmi1*-LacZ mice (Fig. 4d). As for *Bmi1*-YFP reporter mice, we detected LacZ^+^ cells in clusters between sarcomeres and in perivascular locations (see Additional file 9: Figure S7). The number of β − gal^+^ CM detected in the course of the experiment was similar to that for YFP reporter mice at two and 6six months post-TM induction (3.45 ± 0.35 % and 6.45 ± 1.11 %), respectively (Fig. 4d).

### YFP^+^ CM do not derive from Bone Marrow *Bmi1*^+^ cells

**Fig. 6:**
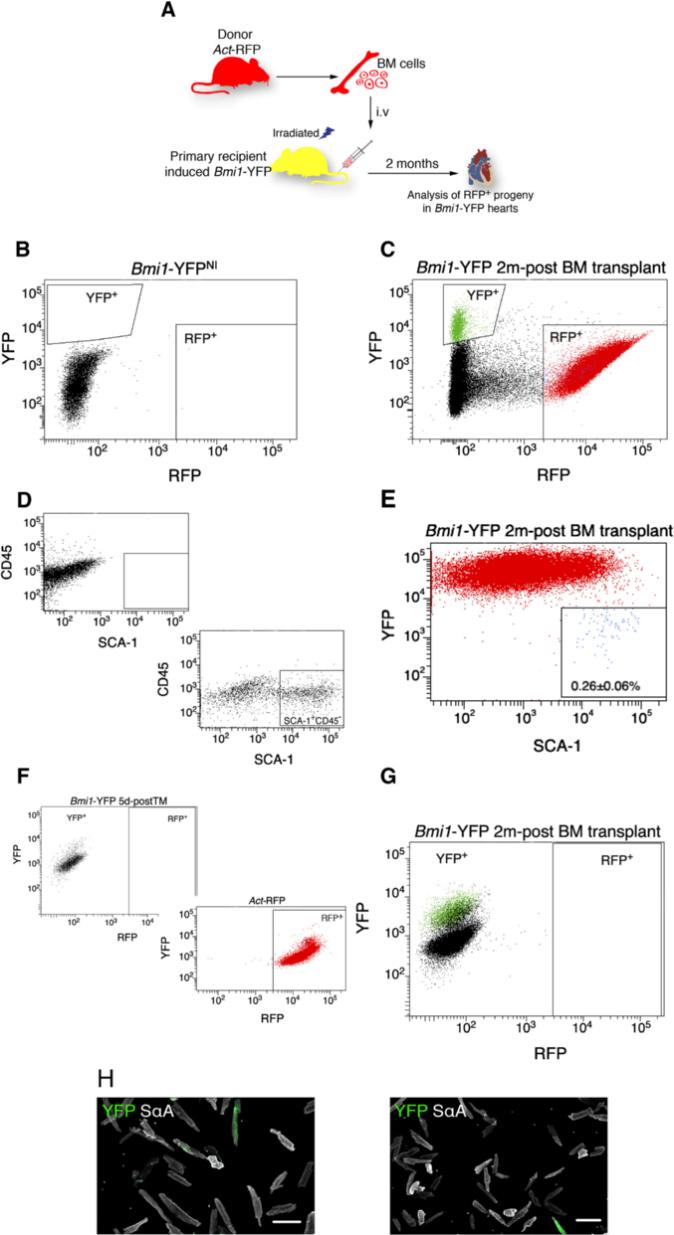
Evaluation of extracardiac *Bmi1* ^+^ cells for *de novo* YFP^+^ CM generation. **a** Scheme for BM (RFP^+^) transplant into a lethally irradiated, TM-induced *Bmi1*-YFP mouse. **b**-**e** FACS analysis of the non-CM fraction of *Bmi1*-YFP mice after BM transplant (*n* = 3). **b** No YFP^+^ or RFP^+^ heart cells were detected in non-induced *Bmi1*-YFP mice. **c** FACS analysis of induced chimeric *Bmi1*-YFP hearts two months post-BM transplant confirmed the presence of YFP^+^ (3.53 ± 0.87 %) and RFP^+^ cells (45.5 ± 15 %). **d** Negative control for SCA-1 and CD45 staining of the non-CM fraction of *Bmi1*-YFP hearts (*left*) and percentage of SCA-1^+^CD45^−^ cells after the staining (20 ± 3.74 %; *right*). **e** Percentage of SCA-1^+^CD45^−^ in RFP^+^ cells in the non-CM fraction of chimeric *Bmi1*-YFP hearts two months post-transplant (0.26 ± 0.06 %). (**f**-**h**) FACS and ICC of the CM compartment of chimeric *Bmi1*-YFP mice two months post-transplant (*n* = 3). **f** No RFP or YFP expression was detected in *Bmi1*-YFP CM analyzed by FACS 5d-postTM (*left*). RFP^+^ CM from *Act*-RFP mice (*right*). **g** No RFP^+^ CM were detected in chimeric *Bmi1*-YFP mice two months post-transplant. **h** ICC of chimeric *Bmi1*-YFP CM two months post-transplant. Bars, 100 μm. Data shown as mean ± SD. *YFP* yellow fluorescent protein, *CM* cardiomyocytes, *FACS* fluorescence-activated cell sorting

Given that *Bmi1*-Cre locus activation in *Bmi1*-YFP mouse is not heart-specific [23, 24] and that bone marrow (BM) cells are reported to contribute with *de novo* CM in heart regeneration [49], we evaluated the potential contribution of BM-derived cell populations to *de novo* CM formation. We transplanted BM from *Act*-mRFP into *Bmi1*-YFP mice (chimeras) (Fig. 6a); recipients were sacrificed two months post-transplant, hearts were dissociated and analyzed by FACS. No YFP^+^ or RFP^+^ cells were detected in non-induced *Bmi1*-YFP mice (Fig. 6b). Two months after BM transplant, non-CM cells from TM-induced chimeric heart confirmed the presence of YFP^+^ (3.53 ± 0.87 %) and mRFP^+^ cells (45.5 ± 15 %) (Fig. 6c). Analysis of heart non-CM cells from transplanted *Bmi1*-YFP mice showed a minimal contribution of SCA-1^+^CD45^−^ cells, estimated at < 0.26 ± 0.06 % (Fig. 6d, e).

Analysis of CM-enriched fractions of *Bmi1*-YFP chimera heart showed YFP^+^ CM, with no mRFP^+^ CM at two months post-transplant (Fig. 6f-h) compared to CM preparations from *Act*-mRFP control mice (Fig. 6f). These results were confirmed by immunocytochemistry in plated CM (Fig. 6h). We observed no fused cells (orange) in CM from chimeric *Bmi1*-YFP hearts (Fig. 6h). The results suggest that, as for other putative cardiac stem cells [18, 50], BM-derived cells do not contribute notably to the B-CPC population, either directly or after fusion with resident non-CM cells.

### B-CPC in the general context of cardiac repair and cardiomyocyte turnover

Cardiac progenitors are routinely isolated based on expression of the stem cell hematopoietic marker SCA-1 [9], although SCA-1 appears to label a heterogeneous population with predominantly endothelial potential [16, 50]. Genetic deletion of *Sca-1* showed that resident CPC do not respond efficiently to pathological damage *in vivo*, consistent with the impaired growth and survival of cardiac progenitor cells *in vitro* [50]. *Sca-1* labels a non-CM population in heart that contributes to CM generation during homeostasis (4.55 ± 0.87 %) [16]. The SCA-1^+^ CPC contribution to the repair process is only found after ischemic damage and pressure overload, but not after acute myocardial infarction. Finally, using the *Rosa26-*Confetti reporter mouse strain, the authors propose limited expansion of *Sca1*-derived clones and limited pluripotential capacity, which suggests that only a small *Sca-1*^+^ cell subset differentiates into the CM lineage [16]. It is tempting to consider that *Bmi1*-CPC, which make up 5.4 ± 0.4 % of SCA-1^+^ CPC, are related to this proposed subset.

Malliaras *et al.* described a transient amplifying cell population, termed cardioblasts (CdB). These cells are defined by expression of SCA-1 and mature cardiac markers such as *Myh6* or SαA but c-KIT^−^, and have multipotent properties. CdB are activated and increase in an experimental model of cardiac injury (AMI). The fact that CdB are a SCA-1^+^ subset, combined with the similarity to our results, strongly suggests partial overlap between these subpopulations. Further studies are needed to fully understand this question.

The classical endothelial marker *Tie-1* was recently reported to define a cardiac progenitor population [19]. These cells (c-KIT^low^) generate 70 % of SCA-1^+^ intermediate perivascular progenitors and contribute to homeostatic CM turnover. Our *Bmi1*-CPC RNA expression profile (unpublished results) showed moderate expression of *Tie-1* and other endothelial driver constructs used by the authors for confirmation [19], as well as high CD31 levels (see Additional file 4: Figure S2A). These data suggest a functional relationship between the two populations. Specific research will be needed to explore the interplay between both populations and their role in the adult endothelium.

All of these cardiac progenitor populations, linked by SCA-1 expression, show partially overlapping similarities with *Bmi1*-CPC. The apparent discrepancies in cardiac progenitor identity and responses nonetheless remain to be resolved. Some of the cardiac stem-like populations identified might represent distinct developmental or physiological stages of a single resident stem cell population [51].

Our lineage tracing analysis shows that high *Bmi1* expression in the adult heart labels a resident population that contributes to CM turnover, suggesting a role for *Bmi1* in heart turnover. Pulsed *Bmi1*^*+*^ cells remained in adult mouse heart for over one year (currently observed to two years of age; not shown), although they appear to decline in aged mice (two- years-old), and probably become more senescent. Gene expression, combined with the non-drastic dilution of the label over this period, implies intrinsic self-maintenance with no apparent contribution of more primitive or extracardiac progenitors to *de novo* CM formation [52, 53] and our findings here.

A number of studies have attempted to identify a cardiac non-stem cell source for adult cardiomyocyte replacement, suggesting that adult CM dedifferentiate and proliferate to give rise to *de novo* CM [6, 7]. In our experimental model using the *αMHC* promoter, we confirmed that at 5d-postTM, a large proportion of CM are labeled (see Additional file 7: Figure S5B,C). This temporal window is also sufficient to ensure clearance of TM, given its estimated 6 h half-life in the mouse, which rules out long-term TM effects [54]. In contrast, in our *Bmi1*-YFP mouse model, we found no indication of Cre expression or activity in the CM population at 5d-postTM. Although we cannot currently exclude additional sources of heart turnover from other stem cell-like populations or a possible functional connection between putative differentiation of rare *Bmi1*^+^ CM, our results support the idea that *Bmi1*^+^ non-myocyte cells (B-CPC) contribute to homeostatic cardiomyocyte turnover in the mammalian adult heart.

## Conclusions

*Bmi1*^*+*^ non-CM cells (B-CPC) form a small fraction of the SCA-1 population; they express high levels of stemness and cardiac lineage specification markers, contribute to the main heart lineages *in vitro,* and are capable of *in vivo* self-maintenance. B-CPC is likely to be a heterogeneous population that contains progenitor cells; further studies are needed for a better understanding of the population and its specific subpopulations. Improved characterization of the biology of these cells will help to define *in vivo* hallmarks as well as potential ontogenic relationships with other adult cardiac progenitor-like populations and to identify the factors needed to harness their potential for effective cardiac cell therapy.

## Additional files

Additional file 1: Table S1. Valiente-Alandi.jpg. Antibodies used in immunodetection. Antibodies used in this study. (JPEG 292 kb) Additional file 2: Table S2. Valiente-Alandi.jpg. Primers used in genomic PCR and qRT-PCR. Primers used in this study. (JPEG 393 kb) Additional file 3: Figure S1. Valiente-Alandi.jpg. Partial comparative expression profile of Bmi1 in SCA-1+ CPC and ES. Comparative *Bmi1* expression and other stemness-related genes in SCA-1^+^ CPC and embryonic stem cells (ES). *Bmi1* expression response to oxygen culture conditions and passage number. (JPEG 843 kb) Additional file 4: Figure S2. Valiente-Alandi.jpg. Flow cytometry analysis in B-CPC throughout mouse lifespan. Flow cytometry characterization of non-CM YFP^+^ cells derived from *Bmi1*-YFP hearts at 5d-postTM and 1y-postTM. (JPEG 926 kb) Additional file 5: Figure S3. Valiente-Alandi.jpg. Representative immunohistology image from Bmi1-tomato mice heart 5d-postTM induction with high tomato background. Distribution of B-CPC cells both in atria and ventricles at 5d-postTM. (JPEG 429 kb) Additional file 6: Figure S4. Valiente-Alandi.jpg. Cardiac explant model. GFP immunostaining of cardiac explants from 5d-postTM *Bmi1*-YFP mice. (JPEG 860 kb) Additional file 7: Figure S5. Valiente-Alandi.jpg. Bmi1 expression and Cre enzyme activity in murine Bmi1-YFP hearts. Bmi1 expression in YFP+ non-CM, YFP- non-CM and CM compartments 5d-postTM and Cre enzyme activity in the CM-enriched-fraction from bitransgenic non-induced controls *Bmi1*-YFP^NI^, *Bmi1*-YFP 5d-postTM and positive control *Myh6*-YFP mice 5d-postTM. (JPEG 728 kb) Additional file 8: Figure S6. Valiente-Alandi.jpg. Calcium transients in freshly isolated YFP+ and YFP- adult CM provide evidence of functional coupling. Contractility and transient Ca^2+^ efflux of freshly isolated YFP+ and YFP- adult CM. (JPEG 513 kb) Additional file 9: Figure S7. Valiente-Alandi.jpg. X-Gal-stained heart tissue of *Bmi1*-lacZ mice 5d-postTM. X-Gal staining showed LacZ^+^ cells in clusters between sarcomeres and in perivascular locations. (JPEG 546 kb)

## Abbreviations

Act-mRFP: Mouse strain that expresses mRFP under the control of the actin promoter
AMI: Acute myocardial infarction
B-CPC: *Bmi1*-expressing cardiac progenitor cells
BMI1: B cell-specific Moloney murine leukemia virus integration site 1
BrdU: 5-bromo-2′-deoxyuridine
CM: Cardiomyocytes
CPC: Cardiac progenitor cells
Cre-ER: Variant of the site-specific (loxP) recombinase Cre that binds to the estrogen receptor module (ER)
CSC: Cardiac stem cells
ES: Mouse embryonic stem cells (R1)
FACS: Fluorescence-activated cell sorting
Fluo-4: AM Non-fluorescent acetoxymethyl ester
GFP: Green fluorescent protein
i.p.: Intraperitoneal
LacZ: Lac Z gene that encodes the *Escherichia coli* β-galactosidase
mRFP: Murine red fluorescent protein
NBRCs: Newborn rat cardiomyocytes
Rosa26: Mouse locus used for constitutive, ubiquitous gene expression
RT-qPCR: Reverse transcription real-time polymerase chain reaction
SCA-1: Stem cell antigen-1
SM: Smooth muscle
SMA: Smooth muscle actin
TM: Tamoxifen
YFP: Yellow fluorescent protein

## References

[1] MacLellan WR, Schneider MD (2000). Genetic dissection of cardiac growth control pathways. Ann Rev Physiol.

[2] Laflamme MA, Murry CE (2011). Heart regeneration. Nature.

[3] Martin-Puig S, Wang Z, Chien KR (2008). Lives of a heart cell: tracing the origins of cardiac progenitors. Cell Stem Cell.

[4] Urbanek K, Cesselli D, Rota M, Nascimbene A, De Angelis A, Hosoda T (2006). Stem cell niches in the adult mouse heart. Proc Natl Acad Sci U S A.

[5] Li TS, Cheng K, Lee ST, Matsushita S, Davis D, Malliaras K (2010). Cardiospheres recapitulate a niche-like microenvironment rich in stemness and cell-matrix interactions, rationalizing their enhanced functional potency for myocardial repair. Stem Cells.

[6] Senyo SE, Steinhauser ML, Pizzimenti CL, Yang VK, Cai L, Wang M (2013). Mammalian heart renewal by pre-existing cardiomyocytes. Nature.

[7] Malliaras K, Zhang Y, Seinfeld J, Galang G, Tseliou E, Cheng K (2013). Cardiomyocyte proliferation and progenitor cell recruitment underlie therapeutic regeneration after myocardial infarction in the adult mouse heart. EMBO Mol Med.

[8] Beltrami AP, Barlucchi L, Torella D, Baker M, Limana F, Chimenti S (2003). Adult cardiac stem cells are multipotent and support myocardial regeneration. Cell.

[9] Oh H, Bradfute SB, Gallardo TD, Nakamura T, Gaussin V, Mishina Y (2003). Cardiac progenitor cells from adult myocardium: homing, differentiation, and fusion after infarction. Proc Natl Acad Sci U S A.

[10] Galvez BG, Sampaolesi M, Barbuti A, Crespi A, Covarello D, Brunelli S (2008). Cardiac mesoangioblasts are committed, self-renewable progenitors, associated with small vessels of juvenile mouse ventricle. Cell Death Differ.

[11] Martin CM, Meeson AP, Robertson SM, Hawke TJ, Richardson JA, Bates S (2004). Persistent expression of the ATP-binding cassette transporter, Abcg2, identifies cardiac SP cells in the developing and adult heart. Dev Biol.

[12] Chong JJ, Chandrakanthan V, Xaymardan M, Asli NS, Li J, Ahmed I (2011). Adult cardiac-resident MSC-like stem cells with a proepicardial origin. Cell Stem Cell.

[13] Steinhauser ML, Lee RT (2011). Regeneration of the heart. EMBO Mol Med.

[14] Rasmussen TL, Raveendran G, Zhang J, Garry DJ (2011). Getting to the heart of myocardial stem cells and cell therapy. Circulation.

[15] Richardson GD, Breault D, Horrocks G, Cormack S, Hole N, Owens WA (2012). Telomerase expression in the mammalian heart. FASEB J.

[16] Uchida S, De Gaspari P, Kostin S, Jenniches K, Kilic A, Izumiya Y (2013). Sca1-derived cells are a source of myocardial renewal in the murine adult heart. Stem Cell Rep.

[17] van Berlo JH, Kanisicak O, Maillet M, Vagnozzi RJ, Karch J, Lin SC (2014). c-kit + cells minimally contribute cardiomyocytes to the heart. Nature.

[18] Malliaras K, Ibrahim A, Tseliou E, Liu W, Sun B, Middleton RC (2014). Stimulation of endogenous cardioblasts by exogenous cell therapy after myocardial infarction. EMBO Mol Med.

[19] Fioret BA, Heimfeld JD, Paik DT, Hatzopoulos AK (2014). Endothelial cells contribute to generation of adult ventricular myocytes during cardiac homeostasis. Cell Rep.

[20] van der Lugt NM, Domen J, Linders K, van Roon M, Robanus-Maandag E, te Riele H (1994). Posterior transformation, neurological abnormalities, and severe hematopoietic defects in mice with a targeted deletion of the bmi-1 proto-oncogene. Genes Dev.

[21] Park IK, Qian D, Kiel M, Becker MW, Pihalja M, Weissman IL (2003). Bmi-1 is required for maintenance of adult self-renewing haematopoietic stem cells. Nature.

[22] Molofsky AV, Pardal R, Iwashita T, Park IK, Clarke MF, Morrison SJ (2003). Bmi-1 dependence distinguishes neural stem cell self-renewal from progenitor proliferation. Nature.

[23] Sangiorgi E, Capecchi MR (2008). Bmi1 is expressed in vivo in intestinal stem cells. Nat Genet.

[24] Sangiorgi E, Capecchi MR (2009). Bmi1 lineage tracing identifies a self-renewing pancreatic acinar cell subpopulation capable of maintaining pancreatic organ homeostasis. Proc Natl Acad Sci U S A.

[25] Zacharek SJ, Fillmore CM, Lau AN, Gludish DW, Chou A, Ho JW (2011). Lung stem cell self-renewal relies on BMI1-dependent control of expression at imprinted loci. Cell Stem Cell.

[26] Lukacs RU, Memarzadeh S, Wu H, Witte ON (2010). Bmi-1 is a crucial regulator of prostate stem cell self-renewal and malignant transformation. Cell Stem Cell.

[27] Biehs B, Hu JK, Strauli NB, Sangiorgi E, Jung H, Heber RP (2013). BMI1 represses Ink4a/Arf and Hox genes to regulate stem cells in the rodent incisor. Nature Cell Biol.

[28] Tanaka T, Komai Y, Tokuyama Y, Yanai H, Ohe S, Okazaki K (2013). Identification of stem cells that maintain and regenerate lingual keratinized epithelial cells. Nature Cell Biol.

[29] Dovey JS, Zacharek SJ, Kim CF, Lees JA (2008). Bmi1 is critical for lung tumorigenesis and bronchioalveolar stem cell expansion. Proc Natl Acad Sci U S A.

[30] Dong Q, Chen L, Lu Q, Sharma S, Li L, Morimoto S (2014). Quercetin attenuates doxorubicin cardiotoxicity by modulating Bmi-1 expression. Br J Pharmacol.

[31] Gonzalez-Valdes I, Hidalgo I, Bujarrabal A, Lara-Pezzi E, Padron-Barthe L, Garcia-Pavia P (2015). Bmi1 limits dilated cardiomyopathy and heart failure by inhibiting cardiac senescence. Nature Comm.

[32] Urbanek K, Torella D, Sheikh F, De Angelis A, Nurzynska D, Silvestri F (2005). Myocardial regeneration by activation of multipotent cardiac stem cells in ischemic heart failure. Proc Natl Acad Sci U S A.

[33] Ellison GM (2011). Torella D, Dellegrottaglie S, Perez-Martinez C, Perez de Prado A, Vicinanza C, et al. Endogenous cardiac stem cell activation by insulin-like growth factor-1/hepatocyte growth factor intracoronary injection fosters survival and regeneration of the infarcted pig heart. J Am Coll Cardiol.

[34] Messina E, De Angelis L, Frati G, Morrone S, Chimenti S, Fiordaliso F (2004). Isolation and expansion of adult cardiac stem cells from human and murine heart. Cir Res.

[35] Garcia-Bravo M, Moran-Jimenez MJ, Quintana-Bustamante O, Mendez M, Gutierrez-Vera I, Bueren J (2009). Bone marrow-derived cells promote liver regeneration in mice with erythropoietic protoporphyria. Transplantation.

[36] Izarra A, Moscoso I, Canon S, Carreiro C, Fondevila D, Martin-Caballero J, et al. miRNA-1 and miRNA-133a are involved in early commitment of pluripotent stem cells and demonstrate antagonistic roles in the regulation of cardiac differentiation. J Tissue Eng Regen Med. 2014. doi: 10.1002/term.1977. 10.1002/term.1977 25492026

[37] Andoniadou CL, Matsushima D, Mousavy Gharavy SN, Signore M, Mackintosh AI, Schaeffer M (2013). Sox2(+) stem/progenitor cells in the adult mouse pituitary support organ homeostasis and have tumor-inducing potential. Cell Stem Cell.

[38] Pelekanos RA, Li J, Gongora M, Chandrakanthan V, Scown J, Suhaimi N (2012). Comprehensive transcriptome and immunophenotype analysis of renal and cardiac MSC-like populations supports strong congruence with bone marrow MSC despite maintenance of distinct identities. Stem Cell Res.

[39] Marban E (2014). Breakthroughs in cell therapy for heart disease: focus on cardiosphere-derived cells. Mayo Clinic Proc.

[40] Ishigami S, Ohtsuki S, Tarui S, Ousaka D, Eitoku T, Kondo M (2015). Intracoronary autologous cardiac progenitor cell transfer in patients with hypoplastic left heart syndrome: the TICAP prospective phase 1 controlled trial. Circ Res.

[41] Makkar RR, Smith RR, Cheng K, Malliaras K, Thomson LE, Berman D (2012). Intracoronary cardiosphere-derived cells for heart regeneration after myocardial infarction (CADUCEUS): a prospective, randomised phase 1 trial. Lancet.

[42] Pfister O, Mouquet F, Jain M, Summer R, Helmes M, Fine A (2005). CD31- but not CD31+ cardiac side population cells exhibit functional cardiomyogenic differentiation. Cir Res.

[43] Smart N, Bollini S, Dube KN, Vieira JM, Zhou B, Davidson S (2011). De novo cardiomyocytes from within the activated adult heart after injury. Nature.

[44] Izarra A, Moscoso I, Levent E, Canon S, Cerrada I, Diez-Juan A (2014). miR-133a enhances the protective capacity of cardiac progenitors cells after myocardial infarction. Stem Cell Rep.

[45] Baumann CI, Bailey AS, Li W, Ferkowicz MJ, Yoder MC, Fleming WH (2004). PECAM-1 is expressed on hematopoietic stem cells throughout ontogeny and identifies a population of erythroid progenitors. Blood.

[46] Blanpain C, Lowry WE, Geoghegan A, Polak L, Fuchs E (2004). Self-renewal, multipotency, and the existence of two cell populations within an epithelial stem cell niche. Cell.

[47] Ilan N, Madri JA (2003). PECAM-1: old friend, new partners. Curr Opin Cell Biol.

[48] Liu J, Willet SG, Bankaitis ED, Xu Y, Wright CV, Gu G (2013). Non-parallel recombination limits Cre-LoxP-based reporters as precise indicators of conditional genetic manipulation. Genesis.

[49] Orlic D, Kajstura J, Chimenti S, Limana F, Jakoniuk I, Quaini F (2001). Mobilized bone marrow cells repair the infarcted heart, improving function and survival. Proc Natl Acad Sci U S A.

[50] Bailey B, Fransioli J, Gude NA, Alvarez R, Zhang X, Gustafsson AB (2012). Sca-1 knockout impairs myocardial and cardiac progenitor cell function. Circ Res.

[51] Ellison GM, Galuppo V, Vicinanza C, Aquila I, Waring CD, Leone A (2010). Cardiac stem and progenitor cell identification: different markers for the same cell?. Front Biosci.

[52] Wagers AJ, Sherwood RI, Christensen JL, Weissman IL (2002). Little evidence for developmental plasticity of adult hematopoietic stem cells. Science.

[53] Loffredo FS, Steinhauser ML, Gannon J, Lee RT (2011). Bone marrow-derived cell therapy stimulates endogenous cardiomyocyte progenitors and promotes cardiac repair. Cell Stem Cell.

[54] Robinson SP, Langan-Fahey SM, Johnson DA, Jordan VC (1991). Metabolites, pharmacodynamics, and pharmacokinetics of tamoxifen in rats and mice compared to the breast cancer patient. Drug Metab Disp.

