# Supplementary figures and images for: Cardiac Bmi1+ cells contribute to myocardial renewal in the murine adult heart
Source: Stem Cell Res Ther. 2015 Oct 26;6:205. doi: 10.1186/s13287-015-0196-9 (PMC4620653; doi:10.1186/s13287-015-0196-9)

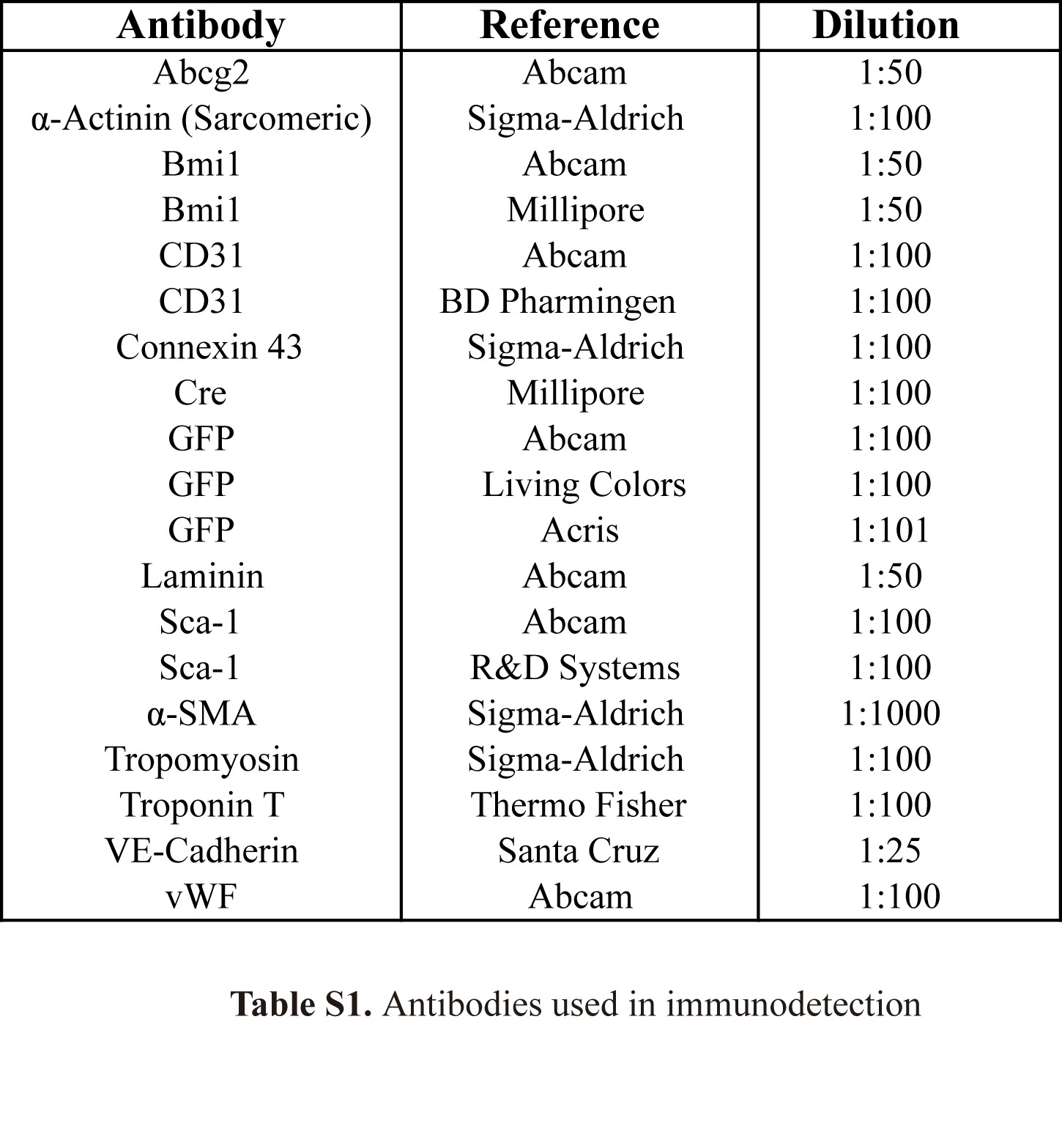

Supplement: Additional file 1: Table S1. — Valiente-Alandi.jpg. Antibodies used in immunodetection. Antibodies used in this study. (JPEG 292 kb) [file 13287_2015_196_MOESM1_ESM.jpg]

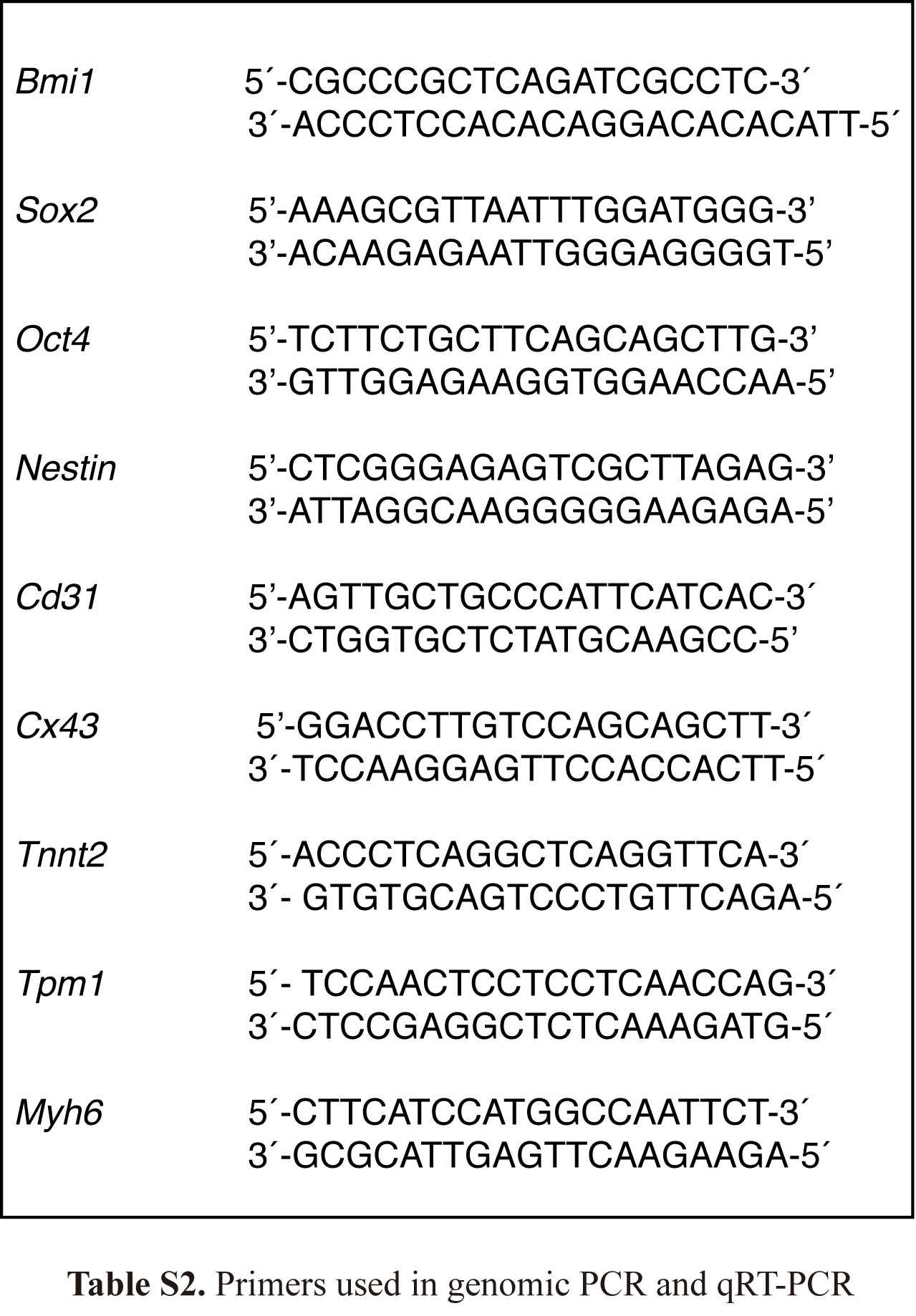

Supplement: Additional file 2: Table S2. — Valiente-Alandi.jpg. Primers used in genomic PCR and qRT-PCR. Primers used in this study. (JPEG 393 kb) [file 13287_2015_196_MOESM2_ESM.jpg]

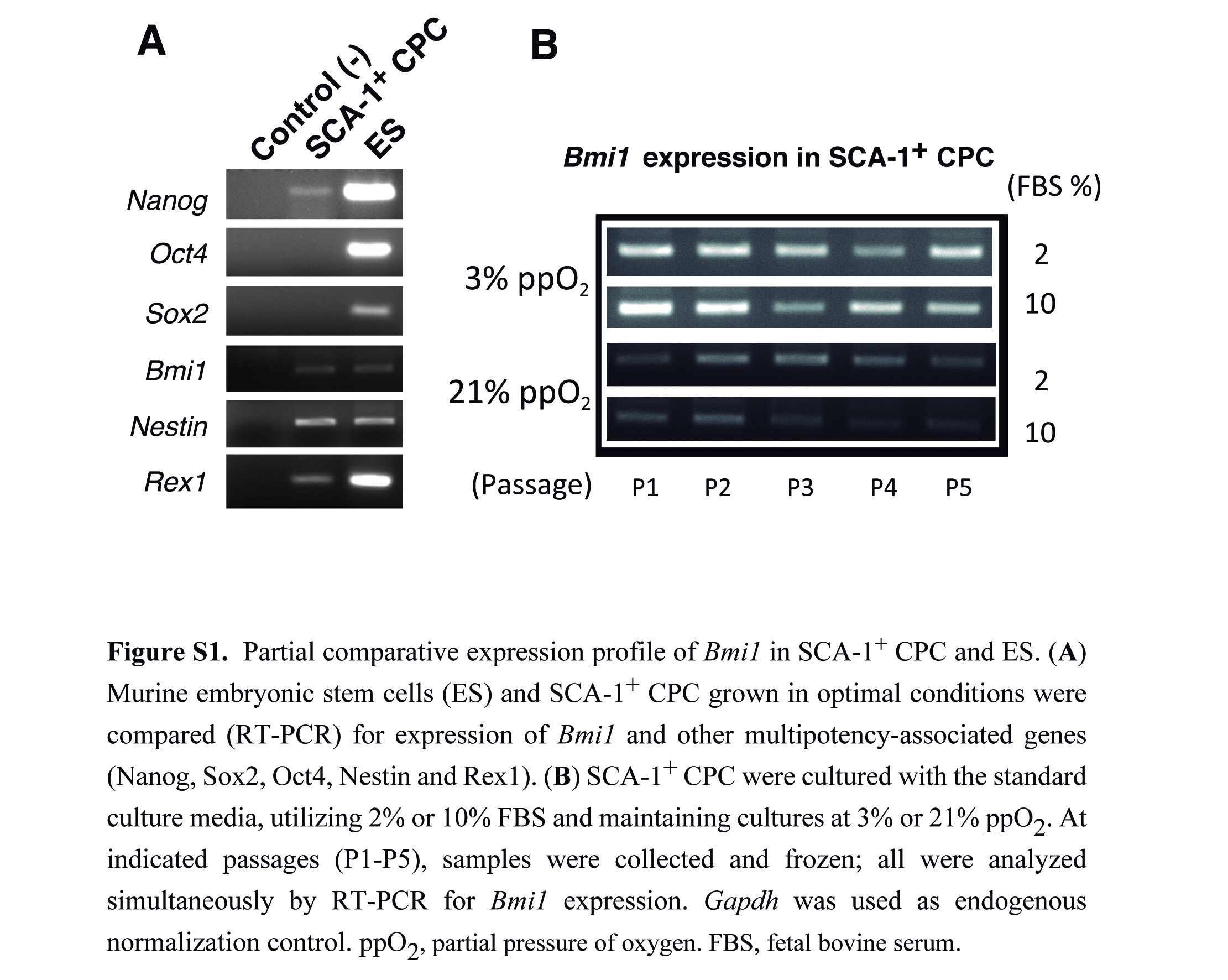

Supplement: Additional file 3: Figure S1. — Valiente-Alandi.jpg. Partial comparative expression profile of Bmi1 in SCA-1+ CPC and ES. Comparative Bmi1 expression and other stemness-related genes in SCA-1+ CPC and embryonic stem cells (ES). Bmi1 expression response to oxygen culture conditions and passage number. (JPEG 843 kb) [file 13287_2015_196_MOESM3_ESM.jpg]

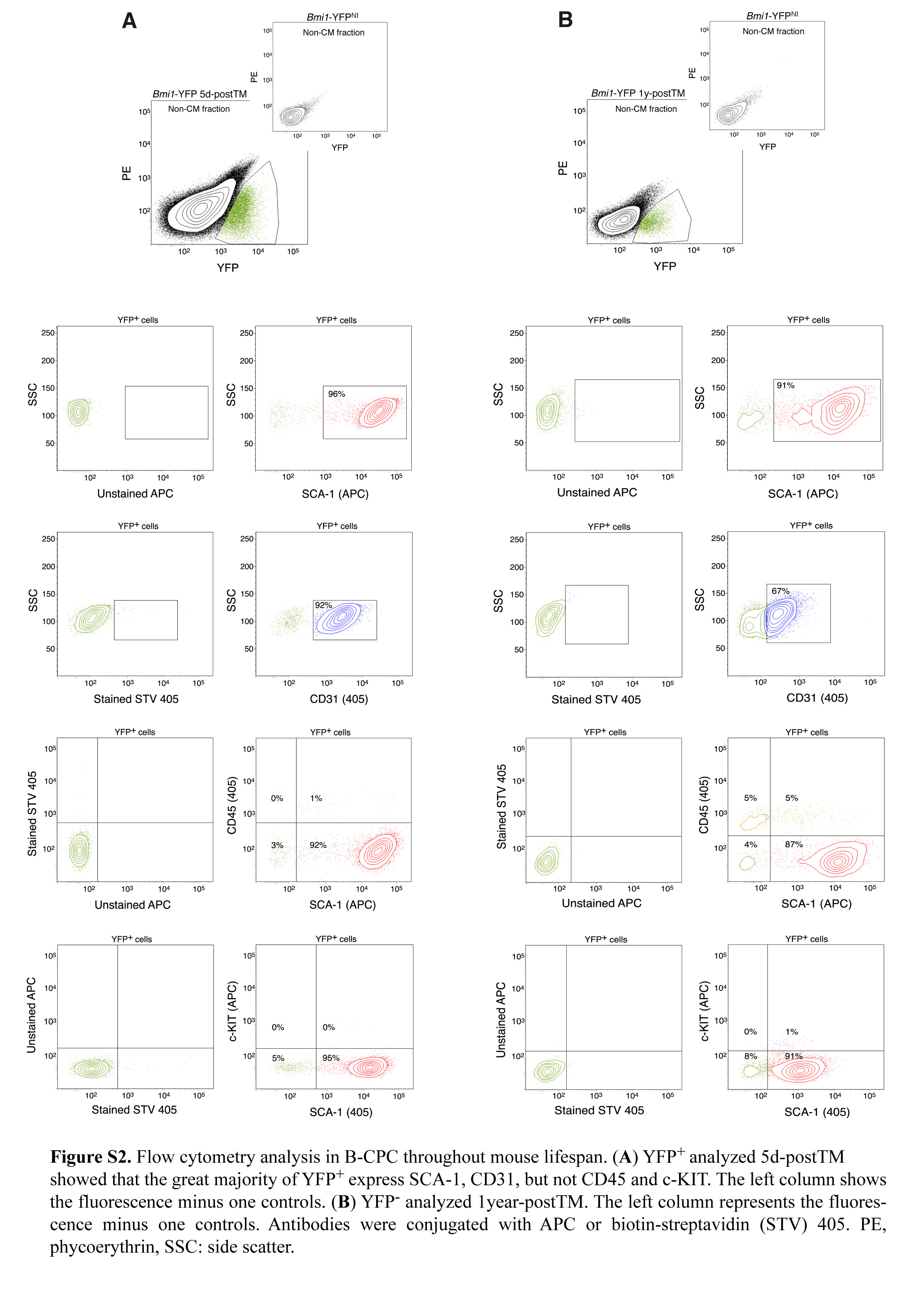

Supplement: Additional file 4: Figure S2. — Valiente-Alandi.jpg. Flow cytometry analysis in B-CPC throughout mouse lifespan. Flow cytometry characterization of non-CM YFP+ cells derived from Bmi1-YFP hearts at 5d-postTM and 1y-postTM. (JPEG 926 kb) [file 13287_2015_196_MOESM4_ESM.jpg]

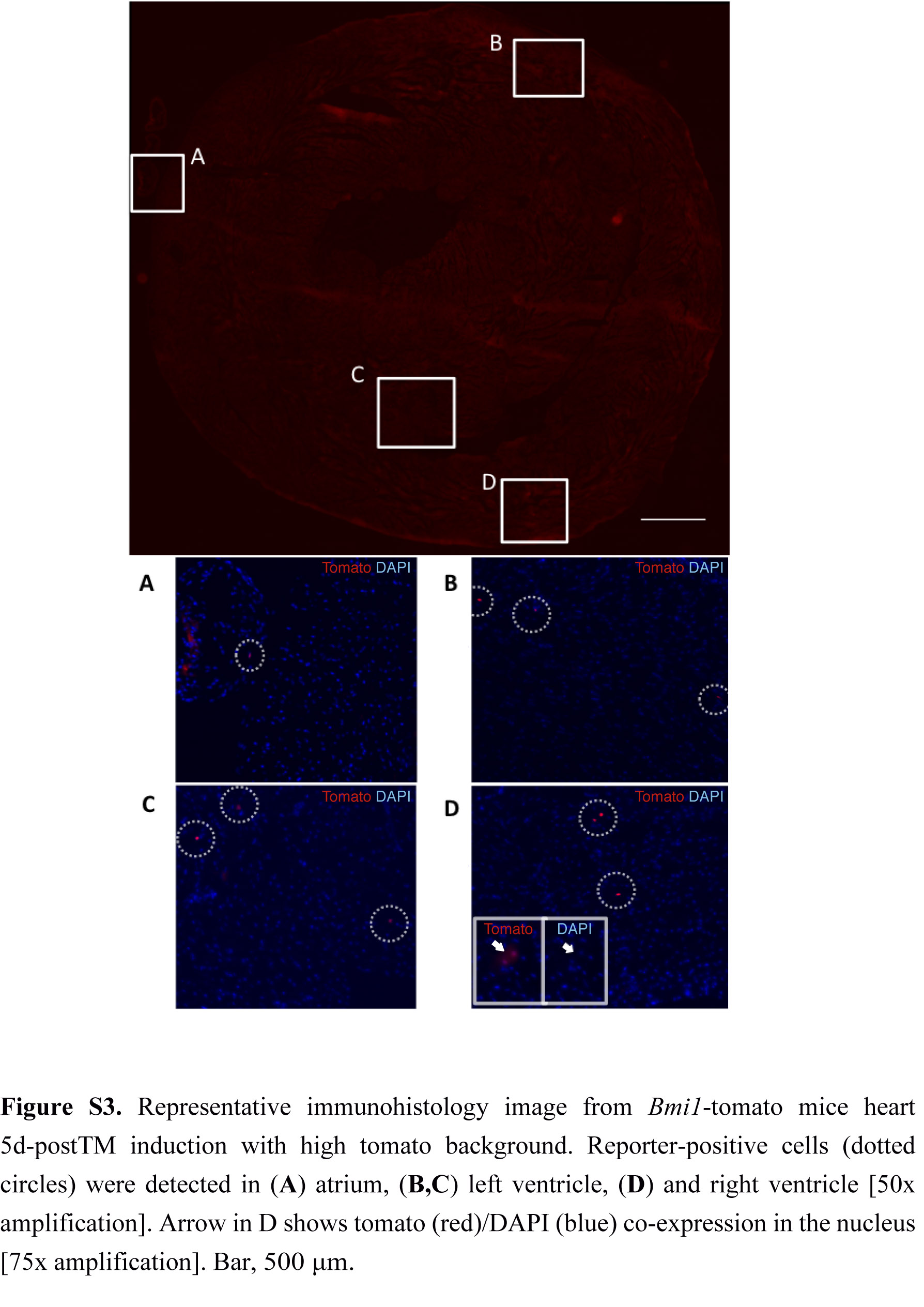

Supplement: Additional file 5: Figure S3. — Valiente-Alandi.jpg. Representative immunohistology image from Bmi1-tomato mice heart 5d-postTM induction with high tomato background. Distribution of B-CPC cells both in atria and ventricles at 5d-postTM. (JPEG 429 kb) [file 13287_2015_196_MOESM5_ESM.jpg]

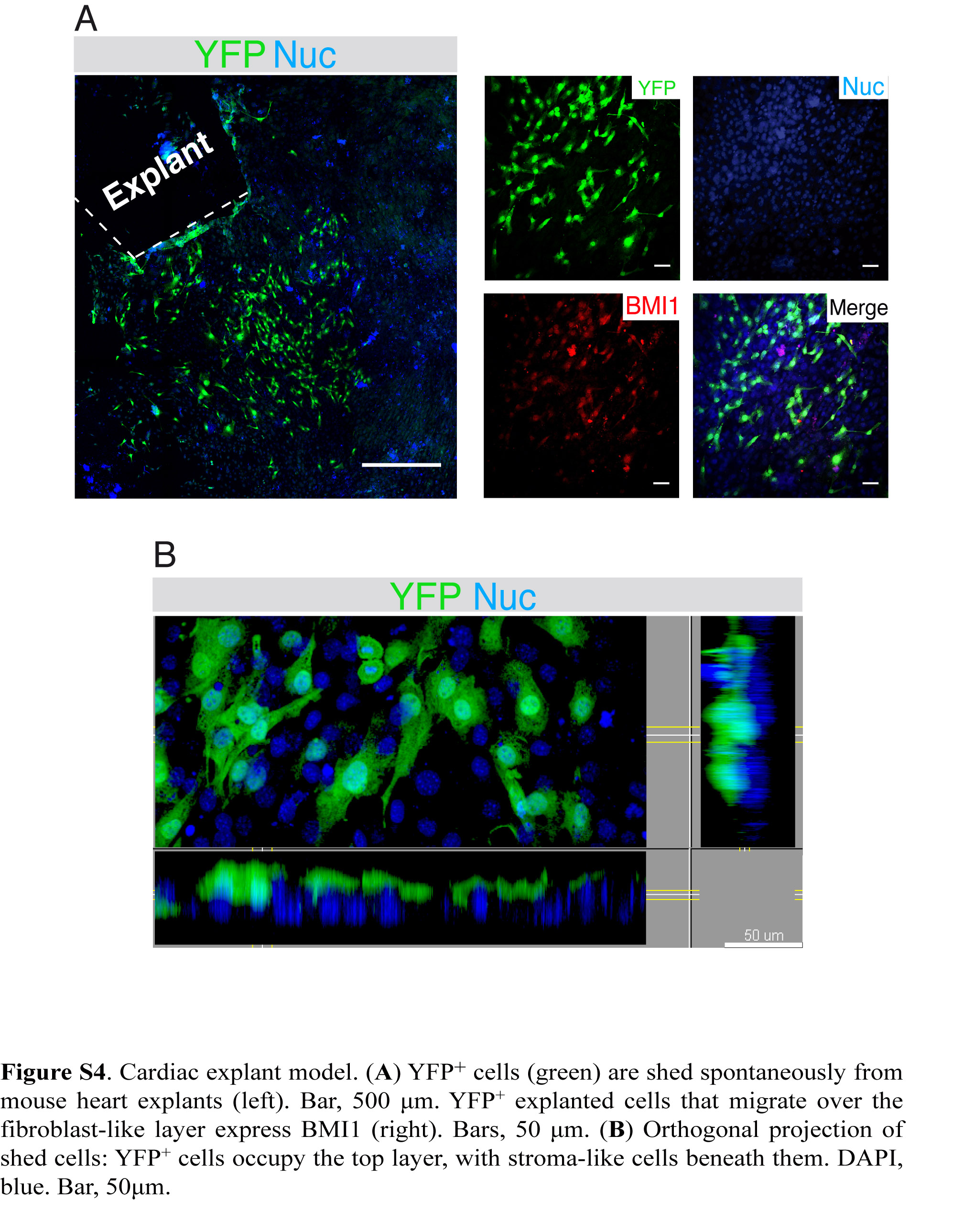

Supplement: Additional file 6: Figure S4. — Valiente-Alandi.jpg. Cardiac explant model. GFP immunostaining of cardiac explants from 5d-postTM Bmi1-YFP mice. (JPEG 860 kb) [file 13287_2015_196_MOESM6_ESM.jpg]

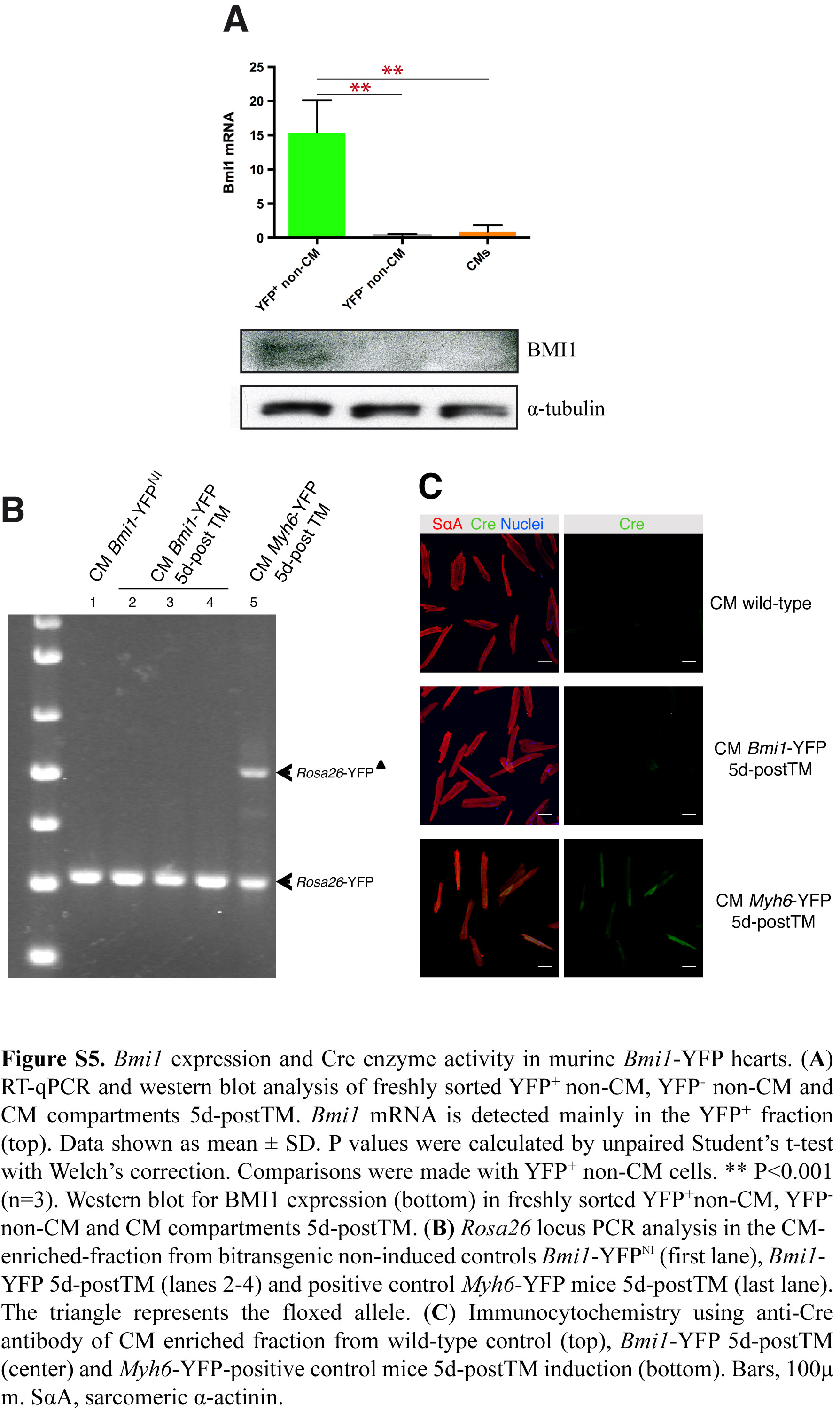

Supplement: Additional file 7: Figure S5. — Valiente-Alandi.jpg. Bmi1 expression and Cre enzyme activity in murine Bmi1-YFP hearts. Bmi1 expression in YFP+ non-CM, YFP- non-CM and CM compartments 5d-postTM and Cre enzyme activity in the CM-enriched-fraction from bitransgenic non-induced controls Bmi1-YFPNI, Bmi1-YFP 5d-postTM and positive control Myh6-YFP mice 5d-postTM. (JPEG 728 kb) [file 13287_2015_196_MOESM7_ESM.jpg]

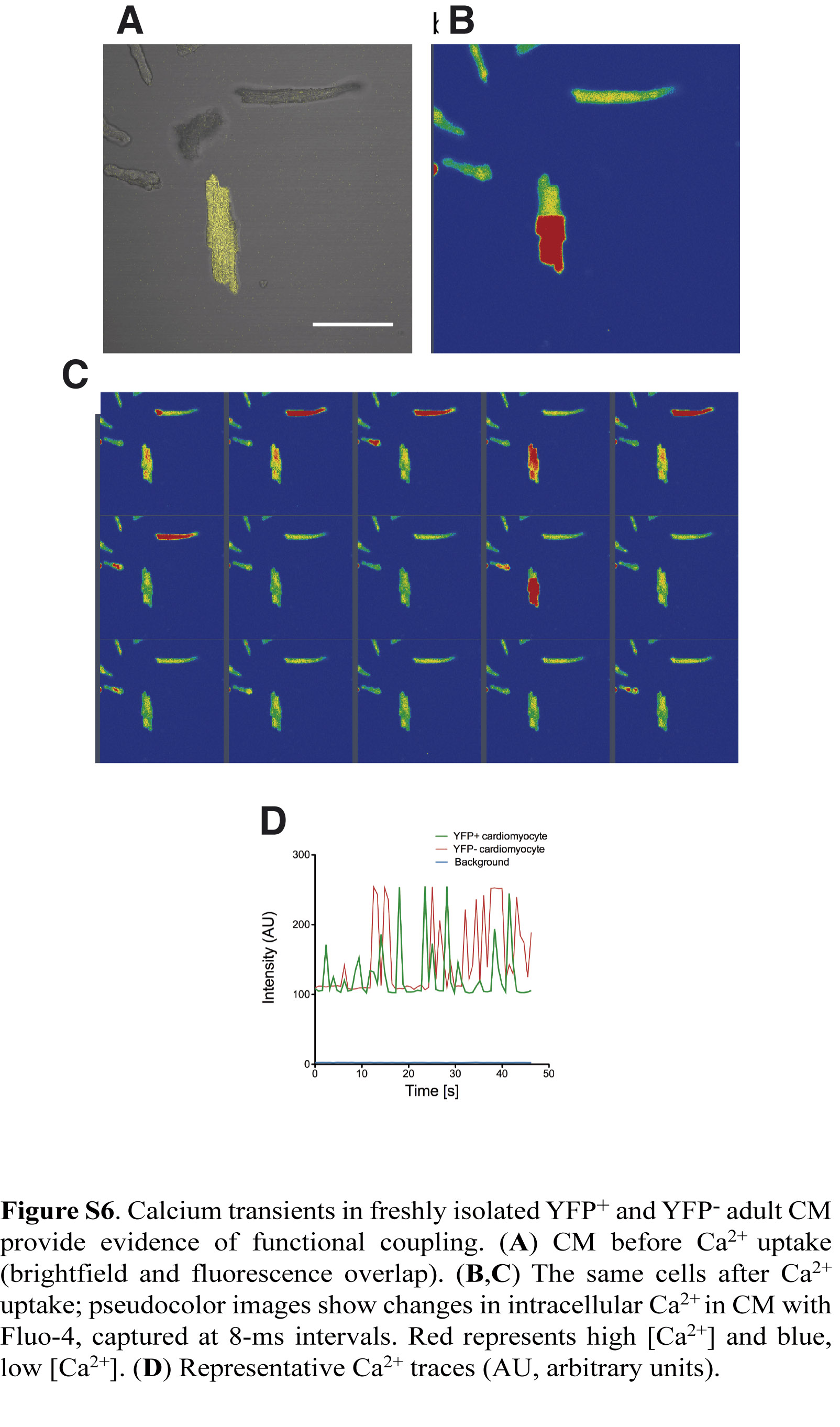

Supplement: Additional file 8: Figure S6. — Valiente-Alandi.jpg. Calcium transients in freshly isolated YFP+ and YFP- adult CM provide evidence of functional coupling. Contractility and transient Ca2+ efflux of freshly isolated YFP+ and YFP- adult CM. (JPEG 513 kb) [file 13287_2015_196_MOESM8_ESM.jpg]

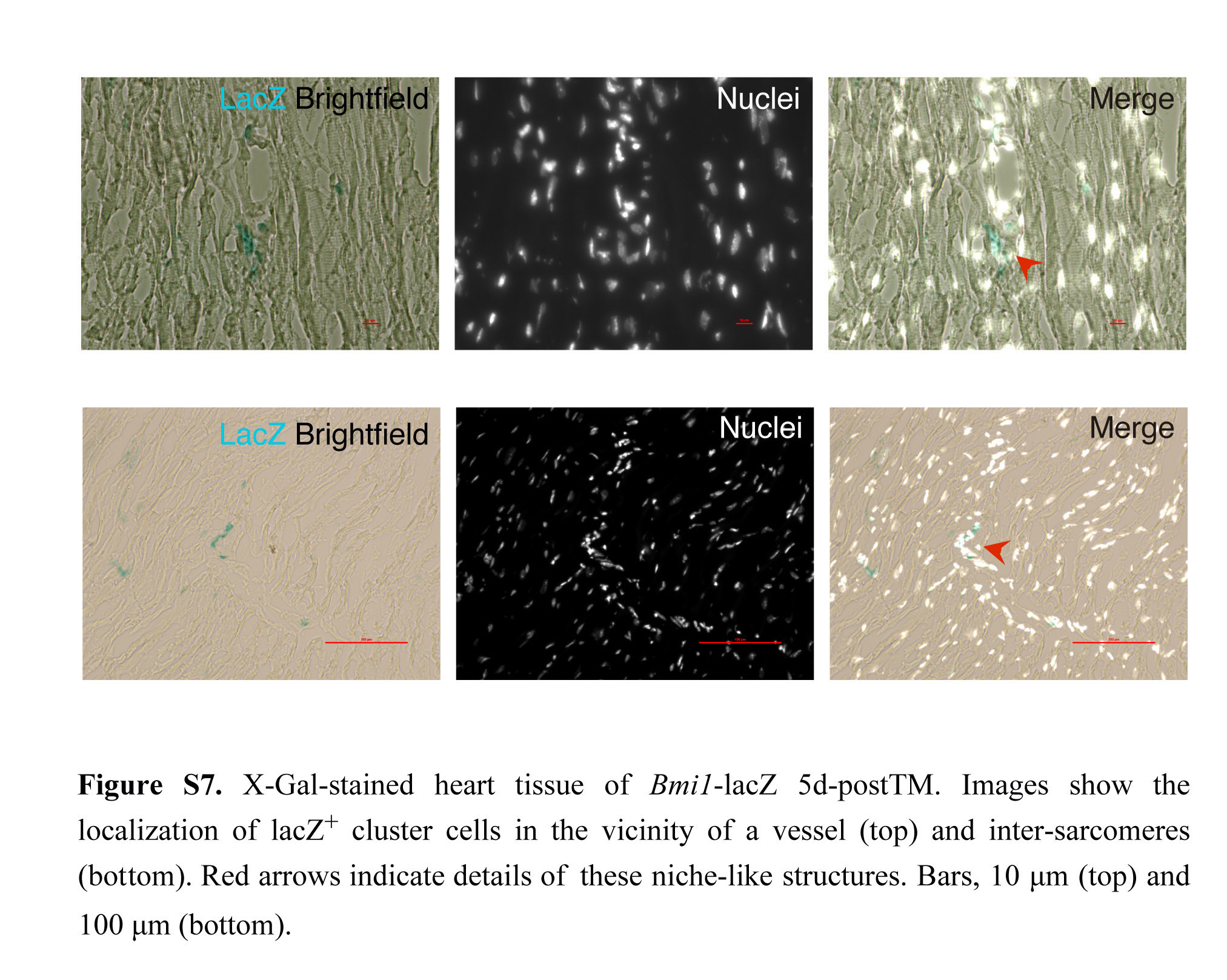

Supplement: Additional file 9: Figure S7. — Valiente-Alandi.jpg. X-Gal-stained heart tissue of Bmi1-lacZ mice 5d-postTM. X-Gal staining showed LacZ+ cells in clusters between sarcomeres and in perivascular locations. (JPEG 546 kb) [file 13287_2015_196_MOESM9_ESM.jpg]
